# Uncovering the Effect of European Policy-Making Initiatives in Addressing Nutrition-Related Issues: A Systematic Literature Review and Bibliometric Analysis on Front-of-Pack Labels

**DOI:** 10.3390/nu14163423

**Published:** 2022-08-19

**Authors:** Marco Francesco Mazzù, Angelo Baccelloni, Piera Finistauri

**Affiliations:** 1 Luiss Business School, Via Nomentana 216, 00162 Rome, Italy; 2 Department of Communication and Social Research, Sapienza University, Via Salaria 113, 00198 Rome, Italy; 3 Department of Business and Management, Luiss University, Viale Romania 32, 00197 Rome, Italy

**Keywords:** front-of-pack label, food policy, nutri-score, NutrInform battery, multiple traffic light, bibliometric analysis, co-citation networks

## Abstract

The last decades have been marked by the introduction of front-of-pack labels (FoPL) as an institutional corrective action against obesity and nutrition-related illnesses. However, FoPL-related policy-making initiatives issued by the European Union evolved over time and led to a diversity of labels with different effects on consumers’ decisions. As a result, the extant literature adapted to the regulative scenario over the years and investigated the effects of the labels, creating consensus on some topics while being fragmented on others. Similarly, policy-makers adapted some regulations to the evidence supported by the research. With the aim to systematize the overall structure and evolution of the literature on FoPL, investigate the presence of a consensus on specific topics through a co-citation analysis, and examine the evolution of the consensus and co-citation networks over the years and potential research gaps, we report the results of bibliometric and co-citation analyses and a systematic literature review involving 170 papers and a selection of 49 articles published in the last months, for a total of 219 articles, analysed according to three timespans (Period 1 (1989–2011); Period 2 (2012–2016) and Period 3 (2017–2022)). Our findings highlight the interplay of policy development and FoPL research, the presence of few self-reinforcing and well-established co-citation networks based on validated evidence in the literature and the presence of alternative emerging theories that offer different and valid perspectives overlooked by mainstream co-citation research networks.

## 1. Introduction

The promotion of nutrition and health benefits is becoming a crucial aspect due to the growth of malnutrition-related issues [1]. Unbalanced dietary habits are the main factor responsible for non-communicable diseases (NCD), overweight and obesity, which are increasingly becoming a serious risk for consumers, oftentimes leading to other more serious illnesses that can be addressed and prevented by implementing measures at the individual, social and governmental levels [1,2].

Among the tools adopted by the food industry and policy-makers, packaging could intensely contribute to changing consumers’ unhealthy habits, nudge them towards salutary choices through informative cues and prevent diseases. However, over the years, the well-known and widespread back-of-pack labels (BoPL) led individuals to a lack of awareness about food features because of the perceived complexity and inaccessibility of such tools [3,4,5,6,7,8].

To this end, governments and public institutions have intervened with the aim of encouraging and nudging consumers towards healthier choices [9].

As a consequence, the main countermeasures activated in the EU, the front-of-pack labels (hereinafter as FoPL), are those tools conceived to provide individuals with the details of the nutritional characteristics of products and improve the diet by increasing the consumption of healthier products [10]. These tools have been conceived to raise consumers’ attention to the nutritional aspects of food products as they are positioned at the front of the package [11]. The FoPL have also been recommended for its efficacy by WHO and is considered a cost-effective tool that would benefit European public health. However, over the years, the absence of a standardized and univocal regulation and the implicit diversity of European states has led to a variety of FoPL.

In a recent attempt to systematize the presence of FoPL in Europe, the EU Commission [12] proposed a framework to interpret the existing categories and differences of such labels (Table 1). More precisely, two broad categories have been identified: (i) nutrient-specific labels, which enlighten consumers on the nutrients of products and (ii) summary labels, which provide a concise overall assessment of the product through letters, colours or numbers. Among the nutrient-specific labels, examples are the NutrInform Battery (NiB) and Multiple Traffic Lights (MTL), while for summary labels, examples in the European context are the Keyhole logo and Nutri-Score (NS).

**Figure 1:**
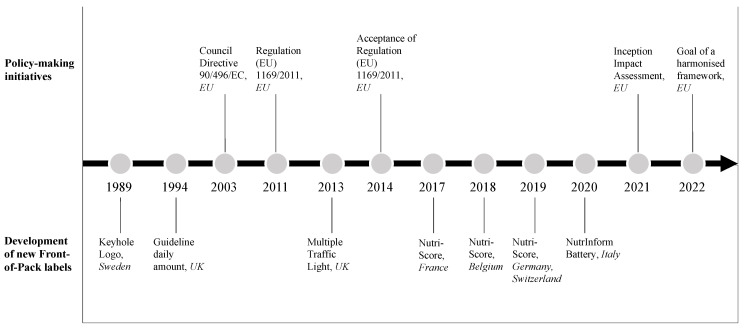
Evolution of policy-making initiatives and FoPL.

The aforementioned labels are the result of a sequence of policy-making and regulatory initiatives, started in the 1990s, that led European countries and Member States to activate institutional corrective and preventive actions against obesity and design their proposals (See Figure 1) to address nutrition-related issues. Indeed, the Council Directive 90/496/EC [13] regulation suggested that all EU Member States require the adoption of labelling systems on food products to be delivered to the final consumer and established the information that a label must contain (such as energy consumption and nutrients such as protein, carbohydrates, fat, fibres, sodium, vitamins, and minerals). A policy-making initiative aimed at aligning the European Union to the best practices available outside the Union in that period in terms of food information provided to consumers [14].

Indeed, the first FoPL, known as the Keyhole logo, appeared in 1989 in Sweden [15], with the aim to prompt individuals to make healthy food choices easier: all the products with a Keyhole logo associated with them represent a healthier option [16].

In 2011, the new EU Regulation 1169/2011 allowed Member States and companies to provide nutrition declarations on the FoPL [17]. In 2014, the application of such regulation became mandatory, enforcing the usage of nutrition declaration on pre-packaged foods regarding the nutritive values in the food [18]. In addition, in 2020, the European Commission modified the previous EU Regulation 1169/2011 [17] focused on the presentation guidelines of nutritional details to consumers by publishing a first impact assessment on nutrition labelling and nutrient profiles on the front of the pack and outlining the initial analysis regarding issues, policy objectives, different solutions and likely impacts [19]. As a result, a set of different proposals has been outlined over the years as effects of the policies implemented and approved by the Member States. In 2013 the Multiple Traffic Light system emerged in the United Kingdom, the Nutri-Score emerged in France in 2017 and in other Member States in the following years: Belgium (2018), Germany (2019), Switzerland (2019) and Luxembourg (2020). In addition, in 2020, the Italian Government proposed the NutrInform Battery System.

With the more recent “Farm-to-Fork” strategy, the EU initiated an attempt to harmonize FoPL while, at the same time, scholars started integrating the concept of FoPL harmonization in their work [20,21].

Additionally, within the Inception Impact Assessment, the EU even declared the goal of finding a unifying solution after assessing the differential performance of multiple FoPLs, such as the Nutri-Score, the NutrInform Battery, and the Keyhole [14].

Over the years, the policy-making initiatives and FoPL implementation evolved into a mutual relationship, and the research testifies to this everlasting exchange, highlighting the reciprocal effects of both.

Indeed, extant literature welcomed the proposals of European States researching the effectiveness of the resulting FoPL and their effects on consumers’ attitudes, choices, and behaviours across countries over the years [22,23,24,25].

The theoretical framework of Grunert and Wills [26] has been one of the most recurrent in the literature [22,23,24,27,28,29,30]. In the theoretical framework [26], they distinguish two types of understanding: objective and subjective. Objective understanding happens when the meaning the consumer attaches to the information on the label represents what is intended to be conveyed by the sender; subjective understanding is defined as the meaning individually attributed to the information on the label by the consumer. Drawing on this framework, many authors attained opposite results. After the release of summary labels, several studies discussed the benefits of the graded indicators (e.g., Nutri-Score) under the investigative lens of objective understanding [23,24,31]. For instance, some scholars observed that summary labels are more effective in conveying information, even if consumers might not fully trust them due to the absence of information related to nutritional values [22,23,24,28]. Conversely, other studies, by focusing on subjective understanding, pointed out that nutrient-specific labels are easier for consumers to understand [32,33]. The degree of healthiness is better understood when the nutrient-specific systems are used because the completeness and specificity of the information allow consumers to better define nutritional values [22].

However, other studies highlight the higher ease of use of colours of the summary labels [25] than FoPL labels showing only numerical information [22]. In addition, a study conducted by Egnell et al. [24] stated that FoPL had an important influence on the understanding of the healthiness of the products, and the NS led to a higher objective understanding relative to other FoPL. Since consumers have limited processing time and need to make decisions quickly, understanding the healthiness is easier with the summary label [32]. While Mejean et al. [34,35] and Van Herpen et al. [36] highlighted that MTL is the FoPL that reaches more consensus thanks to the detailed representation of nutritive values of the product and usage of colours, conversely, when NS labels started to emerge, Ducrot et al. [23] affirmed that the label demonstrates a stronger performance in consumers without nutritional knowledge.

Other studies provide an alternative point of view to previous models. Mazzù et al. [37] developed the front-of-pack acceptance model (FOPAM) and researched how consumers, when exposed to different FoPL, are more likely to accept the information if they perceive the label as useful and easy to use. As a result, the higher the perceived usefulness and ease of use of the FoPL, the greater the acceptance and the likelihood to lead consumers to make healthier and more informed choices [37]. The study offered a different perspective, proving that perceived usefulness and perceived ease of use, when applied to a FoPL-mediated context, are significant predictors of attitude toward FoPL, which in turn predicts behavioural intention toward using FoPL [37]. Specifically, FoPL influence in-store purchases as they inform consumers about the nutrients of the product and are comparable to the decision support system (DSS) that is frequently encountered and evaluated during food shopping [38]. This framework might serve to benchmark FoPL performance in consumer decision-making.

The current literature provides researchers with a plethora of valid and generalizable results. In this prolific field of contributions, some authors seem to converge on the same valid results; other scholars present opposite valid findings, contrasting their demonstrations. While some research streams show a comprehensive analysis of the different perspectives and the diversity of valid findings, other streams mainly refer to convergent contributions, reinforcing the centrality measures of the co-citation networks and the resulting bibliometric indicators. Additionally, many studies confirm different perceptions of product healthiness according to the observed FoPL and with different results in different countries.

This vivid and constant level of contributions lay down the foundations for understanding the multifaceted magnitude of significant effects generated by FoPL. However, to the extent of our knowledge, previous studies did not investigate whether fields of common consensus and over or under-researched topics exist. Additionally, in line with the WHO [39], FoPL are universal and valid tools for fighting diseases. However, the nature, type and magnitude of their effects vary according to the variable investigated, the country, and the type of FoPL observed.

With the aim to understand the structure of the extant literature on FoPL, the fields already covered by researchers, and potential research gaps, in the remainder of this article, we report the results of a bibliometric and co-citation analyses and a systematic literature review involving 170 papers analysed according to three timespans: Period 1 (1989–2011); Period 2 (2012–2016) and Period 3 (2017–2022). The three periods have been defined according to relevant events that marked important steps in the evolution of the policies related to FoPL. In 1989, Sweden developed its first FoPL, and in 1990, the EU issued the first regulation on nutrition labelling systems. In 2011, the European Union authorized Member States and other countries (Switzerland, Lichtenstein, Norway and Iceland) to voluntarily develop FoPLs and contribute to the evidence on the effectiveness of distinct FoPL. From this year onwards, we aim to observe the effects of this regulatory advancement. In 2017, the NS was introduced for the first time in France, and the provision on nutritional information suggested by Regulation (EU) 1169/2011 [17] became mandatory. These events contributed to the EU policy-making environment, and the extant literature varied and registered the effects accordingly.

In this vein, the present research aims to investigate the mutual effects of policies and FoPL and how the extant literature evolved accordingly over the aforementioned periods of time in terms of (i) the presence of a consensus on specific topics through a co-citation analysis, (ii) the evolution of the consensus and co-citation networks over the years, (iii) the relative network indicators and (iv) potential research gaps.

## 2. Materials and Methods

**Figure 2:**
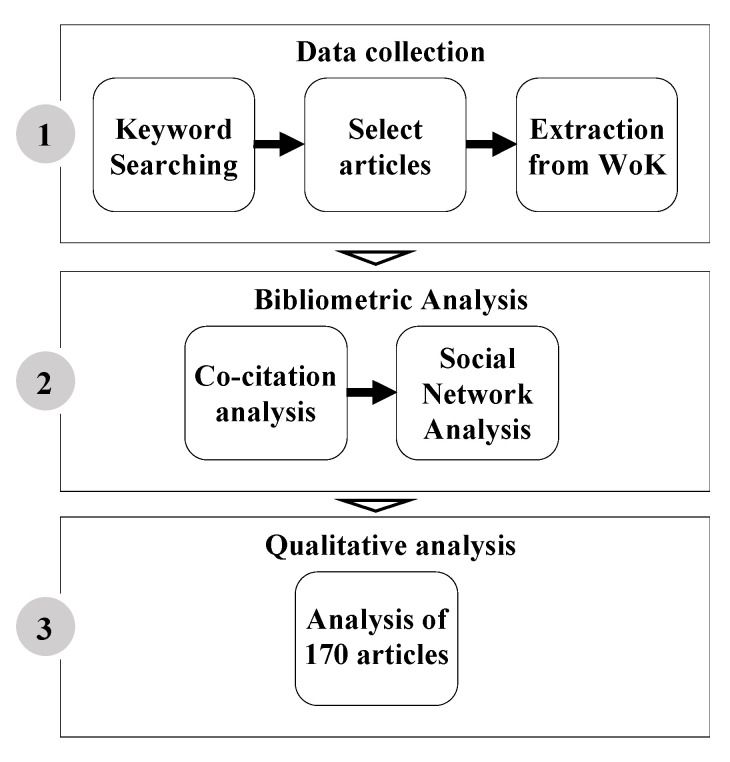
Main stages of the research.

The present study was conducted respecting three sequential stages. In the first stage, we researched articles on Web of Knowledge (WoK) to prepare the data set. In the second stage, we ran a bibliometric and co-citation analysis to further analyse our data. Additionally, we used social network analysis (SNA) to unveil the most influential authors within the network and their characteristics. In the last stage, we systematically reviewed and qualitatively analysed all the articles to investigate shared patterns and topics and assess existing gaps (See Figure 2).

This systematic review has been carried out respecting the guideline of PRISMA and has been registered on PROSPERO.

### 2.1. Data Collection

Considering the broader nature of research on FoPL, the relevant material has been scattered across various journals. In the present study, FoPL articles have been initially retrieved in 9 types of journals: (1) nutrition dietetics journals, (2) public environmental occupational health journals, (3) behavioural sciences journals, (4) economics journals, (5) business journals, (6) communication journals, (7) management journals, (8) food science technology journals, and (9) computer science information systems journals. Since the main goal of this paper was to explore and review the extant literature on FoPL with the aim of understanding the effect of policy-making initiatives and fulfilling potential gaps, the research was mainly conducted focusing on four types of journals. The analysis started with the definition of a query for searching only relevant documents through Web of Knowledge. Authors carried out an extensive search through the keywords “front-of-pack nutrition label”, “front-of-pack”, “labelling”, “nutritional labelling”, “food-package”, “front of pack”, “food label”, “food product”, “nutrition”, “Nutri-Score”, “food marketing”, “NutrInform Battery”, “Keyhole”, “nutrient specific”, “summary labels”, or “Multiple Traffic Light” in the title, abstract and full texts. The adopted timeframe went toward a 32-year research period from 1989 to 2022. Using the “exact phrase” keywords without narrowing the sources, 63,219 documents were reported.

In this perspective, the second step involved a delimitation of sources. Authors recalled only journals in the fields of (1) nutrition dietetics, (2) public environmental occupational health, (3) behavioural sciences and (4) food science technology. This step led to 957 papers, out of which 391 were selected after removing conference proceedings.

**Figure 3:**
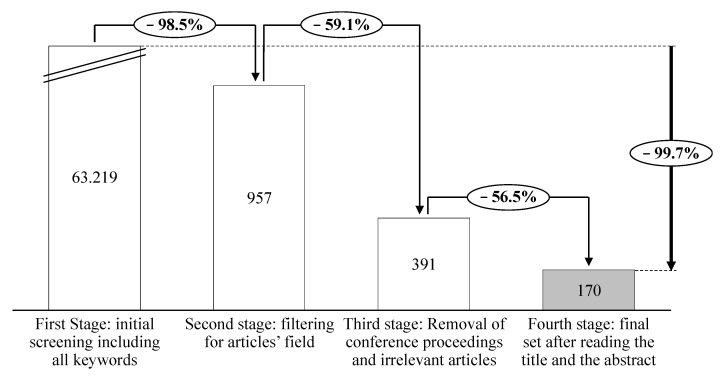
Selection of articles.

Subsequently, after a first read of the titles and abstracts, about 121 papers were excluded because they were considered not concordant with the purposes of the research. This phase led to the selection of 170 articles from 85 journals, which poses the basis of the analysis. Figure 3 summarizes the steps of the article identification process.

### 2.2. Bibliometric Analysis and Centrality Measures

A plethora of business studies leveraged bibliometric analysis to investigate the literature on different topics, including advertising [40], strategic management [41], food labelling [42], and food policy [43]. However, in the nutrition labelling-related fields, bibliometric analysis, especially co-citation analysis, has not been widely used. Crocker et al. [44] reviewed and meta-analysed the literature on FoPL based on 2 years of research. Talati et al. [45] systematically reviewed the literature to assess the consumers’ responses to health claims on FoPL [46]. Aldousari [47] ran a bibliometric analysis to systematize the extant literature on generic health information. However, the latter contribution is not FoPL-specific.

Considering these methodological gaps and with the aim to investigate the structure of the FoPL-related literature, we run a bibliometric analysis and, specifically, a co-citation analysis on the topics. Co-citation analysis is a common bibliometric analysis method [48] and highlights when articles are referenced at the same time in a paper [49]. The higher the level of co-citations, the greater the commonality between two papers [50]. It is a method useful for investigating the cumulative tradition, knowledge base, and intellectual structure of scientific research [51,52]. However, only through qualitative analysis and careful reading of articles is it possible to have a deeper depiction of the contents of the literature [53]. To this end, we systematically reviewed all the articles to further investigate the contents of each article. Previous studies using a combination of co-citation and systematic review indicated complimentary roles of the two analyses [54], and after employing both methods, Åström [55] also indicated that the combination of the two methods provided better results.

Additionally, a set of centrality measures has been employed to discover the role of authors within the network and their relative importance. The betweenness centrality has been computed to discover the times a node is connected to the shortest path between two vertices. The higher the presence of an actor in these paths, the higher the power to control communication since several links are passing through those paths. Nodes that are present on many paths are more relevant in the communication process. Similarly, the closeness centrality has been outlined to represent the average distance, or average shortest path, to all other vertices in the network [56,57]. A central actor would be close, on average, to other vertices in the network. The measure allows evaluating if a central vertex is “close” to other nodes. The sub-components or sub-groups of networks are also called cliques [58], de facto representing co-citation clusters. Moreover, a clique is a component of a larger network where the nodes are strongly linked among them and with a few weak ties with other actors [58].

In the end, the PageRank [59] index was involved to assess the relevance of nodes in the network, modelling the probability that a “random” node that starts at a random position in the network and continues following links will connect to a vertex in the network.

Another node-specific metric was computed to assess the impact of authors in terms of citations. Indeed, each centrality measure was correlated to the H-Index to assess the association between the variables and the impact of an author.

### 2.3. Data Analysis

The bibliometric analysis was carried out with R (bibliometrix package) (Aria et al., 2017). The analysis of the extant literature on FoPL focused on: (1) year of publication; (2) names and the number of authors; (3) the type of study; (4) the analysis carried out; and (5) the co-citation networks and their evolutions over the three timespans. The co-citation networks were developed through the Louvain method [60] for community detection, which aims to maximize the modularity of a subcomponent of a network. By doing so, the density of a subcomponent is higher than the overall density of the entire network, i.e., the nodes connected in a community rely on the highest degree of connections when compared to the degree of connections with the members out of their community and the overall network.

The membership of a specific network is defined on the basis of the links that exist between one author and another, i.e., the number of times they are cited. The authors who mention each other the most belong to the same component of the network; otherwise, they refer to different clusters. In the remainder of the paper, we will number each observed network as co-citation networks 1, 2, 3, 4 and 5. To further observe the mutual effects of the policies on FoPL and vice versa, we investigated the evolution over the time of the literature and divided the dataset according to three timespans: (1) Period 1 (1989–2011); (2) Period 2 (2012–2016); and (3) Period (2017–2022). The results of the aforementioned section are highlighted in the “Quantitative Findings” section.

After observing the quantitative results, we read and analysed all the articles according to the three timespans to find additional evidence emerging from the contents discussed by the authors. The latter are discussed in the “Qualitative Findings” section.

## 3. Results

### 3.1. Quantitative Findings

**Figure 4:**
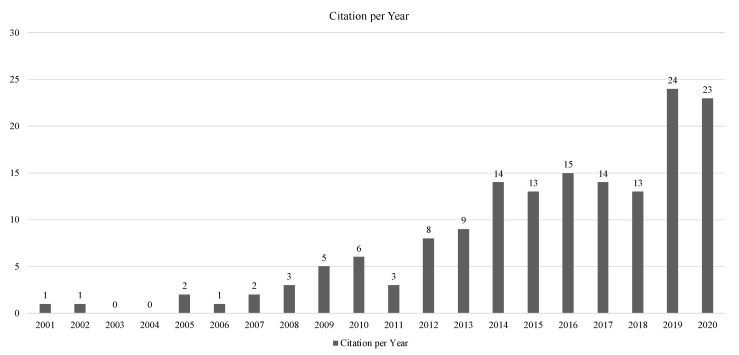
Evolution of the literature over the time.

The 170 papers analysed were collected from 85 journals. Since the timeframe goes from 1989 to 2022, an increasing focus on the topic over the years has been noticed, with two recent peaks of interest in 2019 and 2020 with an average annual growth rate equal to 14.25% and an average citation rate per year of 2.79% (Figure 4).

Of the abovementioned 170 papers, 148 were empirical studies, 3 were research papers and 19 were literature reviews.

**Figure 5:**
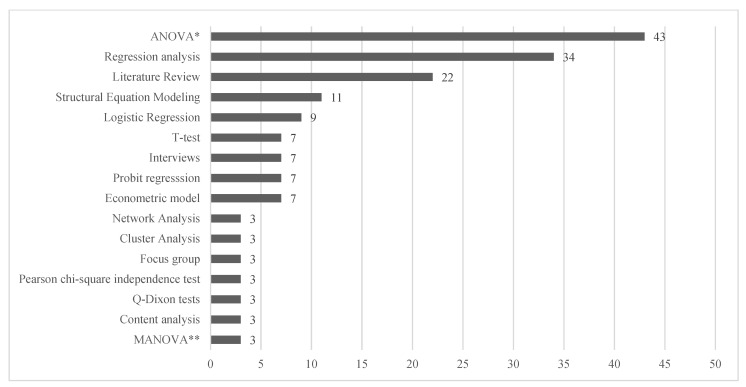
Adopted methodologies and analysis approach. * ANOVA: Analysis of Variance; ** MANOVA: Multivariate Analysis of Variance.

Across the empirical studies, several methodologies have been adopted. As shown in Figure 5, the most used methodologies are ANOVA, regression analysis, literature reviews and structural equation modelling. Only a few authors adopted mixed and qualitative methods to conduct their studies.

Additionally, the analysis involved 485 authors, with an average of 2.85 authors per paper and only 12 single-authored documents. On average, each document was cited 19.14, and the total amount of references reported by all documents in the dataset equals 5234.

The most-cited article was written by Drichoutis et al. [61], followed by Andrews et al. [62] and Barreiro-Hurlé et al. [9] (see Table 2).

**Figure 6:**
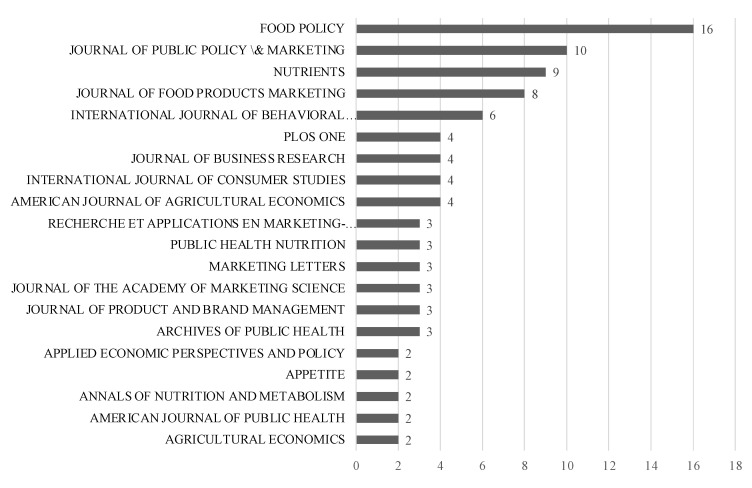
The most-cited sources.

**Figure 7:**
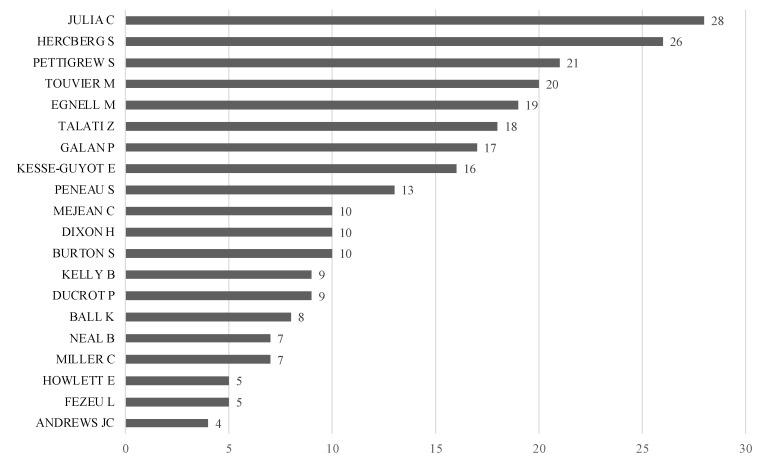
The most-cited articles.

The most relevant journals on the topic are Food Policy, Journal of Public Policy & Marketing, Nutrients and Journal of Food Products Marketing (see Figure 6). Similarly, the most cited authors are depicted in Figure 7.

The most prolific countries are the USA, with 194 documents and 1115 citations, France, with 155 documents and 658 citations, and Australia, with 127 documents and 371 citations. Similarly, the most collaborative countries are the USA, France and Australia (Table 3).

### 3.2. Co-Citation Analysis and Qualitative Findings

Subsequently, we carried out a co-citation analysis divided into the three periods (1989–2011, 2012–2016 and 2017–2022) to further explore whether relevant associations exist among authors.

A total of 5 co-citation networks appeared. In the first analysed timeframe (1989–2011), two co-citation networks emerged, representing the first contributions to FoPL and a sequence of articles published after the introduction of the Keyhole Logo in 1989.

Period 1989–2011: Discovering the relevance of nutrition-related information

This first period is characterized by the first European regulations on the subject of nutrition labels. In 1990, in fact, the European Commission proposed a regulation on information for packaged foods. Then, in 2011, with the “Regulation (EU) No 1169/2011” [17], the topic of FoPL was introduced in a definitive way, without, however, establishing a mandatory use. In this first period, we mainly found two streams of studies (see Supplementary Materials Figure S1): (i) the co-citation network 1 (in red in Supplementary Materials Figure S1), oriented towards the drivers of misinterpretation of labels and the knowledge of consumers and (ii) the co-citation network 2 (in blue in Supplementary Materials Figure S1), focused on the relevance of providing nutritional information through labels and their effects on healthier choices. The width of the line depicts the weight of the links between two nodes. The higher the width, the greater the number of times two authors cite each other. In co-citation network 1, the authors who share the greatest numbers of links are Chandon and Kozup, Balasubramanina and Moorman, Cacioppo and Kozup, Grunert and Kozup, Kozup and Garretson, and Cacioppo and Mitra. In co-citation network 2, the main links are those between Parmenter and Nayga and Gracia and Nayga.

#### 3.2.1. Co-Citation Network 1

In this period, EU consumer policies on FoPL started to emerge and were mainly aligned with the so-called “information paradigm” [14]. According to this paradigm, the higher the level of information on foodstuff provided to consumers, the higher the awareness about the item they purchase and the likelihood of nudging buyers toward healthier choices [14]. The main disadvantage of these assumptions is that they do not properly recognize the consumers’ bounded rationality and consider them perfectly rational in their decision-making process. However, from the regulators’ point of view, individuals are autonomous, dislike limitations and are able to make decisions on their own [14]. This is a feeling even grounded in the European Court of Justice [63], which observes consumers as perfectly reasonable, well-informed, adequately circumspect and able to read and process information in the proper way.

The first attempt at EU regulation on FoPL, Council Directive 90/496/EEC [64], leveraged the aforementioned assumptions, pointing out the relevance of supporting citizens’ decisions with appropriate information on their diets. According to this Recital, the information paradigm has been respected.

In this period of time, the authors belonging to such network in terms of co-citations [65,66,67,68,69,70,71,72] defined the early stage of the research in nutrition labels and offered a series of contributions to investigate the comprehensibility of labels and the consequent ability of the consumer to understand and recognize them (Table 4 and Table 5).

At the very beginning of this stream of studies, Burton et al. [70,71,72] highlighted the complexity of selecting a label that is easy for consumers to understand and showed how nutritional knowledge interacted with nutritional value in such a way that consumers with higher knowledge have a higher likelihood of purchasing a product with better nutritional value [70]. In a subsequent work, he argued that the acceptance of the information varied according to the format of the presentation of the label and stressed the need for further educational efforts to explain the information on new labels (Burton et al. [71]. In contrast, Keller et al. [69] confirmed that the information normally contained on labels does not influence consumers’ nutritional beliefs. This means that consumers prefer the presence of nutrition claims on the front of the package and an independent and detailed verification of nutritional values [69].

Roe et al. [67] conducted a study on this topic and stated that nutrient and product health information has a significant effect on information processing and product evaluation.

In particular, when information is clearly displayed, it is an important tool in qualifying misleading impressions from nutrient content claims [57]. Subsequently, other authors [64,67,68,69,70] added that in helping to remedy potential misinterpretations, the role of information, the level of nutritional knowledge, and the type of advertising claims used become important [64]. The use of elements contained in the disclosure would be useful in correcting potentially misleading omissions, as high nutritional levels may be perceived in a positive way in conjunction with recommended total daily recommended values [4]. Furthermore, it results in a more positive effect to use nutritional claims rather than less specific claims [66]. Conversely, according to Burton et al. [72], many consumers seem to be able to understand nutritional information correctly and evaluate products in the best possible way, thanks to it.

In 2008, the EU Commission developed a proposal [73] and recognized the need to strengthen FoPL as tools able to fight obesity and to improve their effectiveness. This led to the modification of extant FoPL.

In this regard, a parallel stream of research became relevant in the FoPL literature in the following years, proposing a model to assess the antecedents of the acceptance of nutritional information.

Indeed, Grunert [26] revealed the complexities of nutrition labelling and the difficulties consumers find with elements on labels, such as technical terms and numerical calculations. In general, consumers understand the information on the product label, both in relation to individual nutrients and overall healthiness. Greater understanding is shown among consumers in the UK, Sweden, and Germany. Focusing on the UK, a greater understanding emerges overall since the UK has been at the forefront of FoPL in Europe. This is another reason why much research has focused on this background [26]. Grunert et al. [26] continued researching the topic with other authors in 2010 and further added that most respondents had no difficulty in understanding the nutritional information in the FoPL and using it to make deductions about the healthiness of products [74].

#### 3.2.2. Co-Citation Network 2

In this co-citation network, the authors mainly focused on the importance of using labels to make consumers more health-conscious and the relevance of providing truthful information (Table 6 and Table 7). Similar to the co-citation network 1, these authors belong to the early stage of the literature on FoPL and seem positioned in a causal relationship with Directive 90/496/EEC [64], which introduced nutritional labelling without a high awareness of its effectiveness. The research, in this period of time, shed light on the main effect of the labels and supported the evolution of regulations.

The first contribution comes from the study conducted by Ippolito and Mathios [75], which states that a focus on increasing the truthful of information flow is necessary for a good consumer policy.

In addition, Levy et al. [76] argued that the nature of the information and the way it is presented affect the understanding and preference of the labels, evidencing that the most impactful format features lower decision-related efforts required by a situational task. Gracia et al. [77] confirmed this evidence by stating that consumers’ product perceptions differ according to the way the information (facts or claims) and the type of information (nutrition or health) are presented.Furthermore, the research conducted by Teisl et al. [78,79] asserted that the adoption of an information campaign to educate consumers together with the labelling of products to highlight their nutritional characteristics are important to prompt consumers toward healthier choices. Loureiro et al. [80] demonstrated that information about the nutritional content of food products through nutrition labels can make consumers more informed and thus help them to make healthier food choices and follow healthier diets. The research conducted by Gracia et al. [77] confirmed this evidence, explaining a positive link between nutritional labels and healthier choices.

Other authors [75], a few years before, outlined a distinction between the information on the nutrition panel and front-of-the-pack information, demonstrating that the nutrition panel on the back of a pack may provide much useful information when the consumer is interested but may be relatively ineffective in generating consumer interest.

In subsequent research, the importance of knowing the factors that determine consumers’ use of food labels to improve the effectiveness of food labelling policies emerged.

Authors found that different factors affect the usage of labels, such as household food expenditure, income, household size, education, the race of the head of household, urbanization and region of residence [81].

Wansink et al. [82] added that people show a more positive attitude towards short indications than long ones on FoPL, while Wang et al. [81] argued that consumers are positively influenced by more descriptive and informative labels.

Similarly, Wansink et al. [82] confirmed that descriptive labels influence consumers’ intention to repurchase the labelled food directly and indirectly through the overall evaluation of the labelled food.

Finally, concerning the willingness to pay for the product, other research [83,84] shows that subjects’ willingness to pay is higher for products with nutritional information than for products without nutritional information. Moreover, the research suggested that product evaluation is affected by the quantum of nutrition appearing on the label.

In the first two co-citation networks, the interplay between the literature and the regulations clearly appears. The first is aligned to the regulations and the latter, in this period of time, guides the research. In addition, consumers in this period started to be aware of labels and able to understand the nutritional information that informs their decisions. Co-citation network 1 is focused on the investigation of what drives the understanding of consumers, whereas co-citation network 2 is mainly focused on the antecedent of the usage of nutritional labels.

##### Period: 2012–2016—The Drivers of Acceptance of Front-of-Pack Labels

In the second timeframe (2012–2016), the first effects of regulations and new FoPL introductions (such as Guideline Daily Amount, MTL, Fact Up Front and NS) appear. Two main co-citation networks are highlighted, and new authors appear, while other established authors remain (see Supplementary Materials Figure S2). Co-citation network 3, in red in Supplementary Materials Figure S2, is mainly focused on the 5-Color Nutrition Label (today known as Nutri-Score), while co-citation network 4, in blue in Supplementary Materials Figure S2, is mainly focused on all other labels that have emerged over time. The strongest links in co-citation network 3 are those between Julia and Hercberg, Julia and Mejean, Rayner and Bialkova, Julia and Cowburn, Van Herpen and Borgmeier and Mejean and Cowburn. In network 4, the strongest links appear between Grunert and Moorman, Casweel and Moorman, Roberto and Grunert, Kozup and Wansink.

#### 3.2.3. Co-Citation Network 3

With Regulation 1169/2011 [17], the EU required the mandatory provision of nutrition information on packaging to support consumers’ decisions and defeat obesity-related issues. Additionally, the Recitals recognize the need to make FoPL appealing to the average consumers and suggest the need for easy and recognizable labels to facilitate the assessment of nutritional values [83]. This was an advancement that reflects the evolution of the policies and the implied paradigms. Beyond the classic “information paradigm” and the active role of FoPL in the Proposal outlined in 2008, Regulation 1169/2011 [17] laid down the foundations for a central role of FoPL in the fight against obesity and their roles in orienting and affecting consumers’ choices. The FIC Regulation allows Member States and operators to try their own proposals to help consumers. As a result, the literature in this period welcomed many studies about the new FoPL designed by Member States. In this test phase, the research is divided into two main streams: one focused on the NS and one based on the comparison of other labels.

Co-citation network 3, indeed, mainly focuses on the development of the NS label and the ease of use of the information given to consumers through labels (Table 8 and Table 9). One of the first studies, subsequent to EU Regulation 1169/2011, was conducted by Signal et al. [84], which discussed the benefits provided by FoPL to consumers, including the provision of simple and easily understandable information intended as tools able to communicate information about the nutrients and healthiness [36]. In addition, Schwartz et al. (2012) posited that FoPL improved the accuracy of judgement about foods.

In general, drawing on these authors, Ducrot et al. [23] stated that the presence of labels makes it easier for consumers to classify products according to their nutritional quality. However, such labels help to inform consumers but do not influence their choices and thus change their perceptions of health, taste, and purchase intention [85]. In turn, a good FoPL should at least be able to encourage food manufacturers to reformulate products [84,86].

In addition, other potential limits of FoPL outlined in this network are the lack of agreement on the labelling format, limited evidence on which to base this decision, potential opposition from the food industry, conflict of values between the food industry and public health, etc. [84].

Furthermore, other authors explained the relevance of individuals’ boundaries that lead to reduced usage of FoPL. Bonsmann et al. [86], for instance, added that the lack of attention and the lack of motivation toward FoPL’s usage is due to the fact that organoleptic features of food are the main reasons for choosing products. Consumers are influenced by a priori familiarity much more than by the use and effect of labelling schemes [87].

Additionally, the level of detail depicted in a FoPL is highly relevant for final acceptance [88]. Labels with a lot of information are likely to convince more consumers since they offer in-depth details about the product [89].

FoPL labels showing numerical and text information are more difficult for consumers to understand [22]. In contrast, FoPL based on colours and graded indicators seem less complicated [34].

However, the acceptance of FoPL labels varies on a scale according to the overall liking, understanding, attractiveness and the perceived cognitive workload [34]. For instance, it has been proved that graded summary labels, such as the more recent NS (in that period known as NutriNet-Santé or 5-Color Nutrition Label), are easier to understand [23,90]. The label represents a coloured scale (from green to red) to indicate the goodness of the balance of nutritive values [90] and summarizes the nutritional quality of the food or beverage. According to Julia et al. [91,92], this makes it possible to properly discriminate the nutritional quality of foods at various levels and prompts individuals toward the choice of healthier products. In addition, this label seemed to positively influence consumers’ eating habits and their quality of life [93]. Ducrot et al. [23] confirmed that the 5-CNL label, according to consumers, was the easiest label to understand and highlighted a greater efficacy in consumers with no nutritional knowledge.

This co-citation network reflects a stream of studies based on the investigation of the NS label and its effects. While in the other networks, there is a heterogeneity of topics, in this network, over the analysed timespan, there is a clear convergence on the topic and the country of origin of the authors, which links to the national perspective of adoption of specific labels.

#### 3.2.4. Co-Citation Network 4

As opposed to the previous network, the present composition of the literature is dominated by a fragmentation of the contributions, and a clear heterogeneity of the topics/labels investigated (Table 10 and Table 11). A set of articles that compare the effectiveness of other FoPL emerged in the second period, such as the Smart Choices (SM), Multiple Traffic Light and Health Logo.

In addition, many authors from the co-citation network 2 of the timespan 1989–2011 now belong to this one [94,95,96,97,98].

Some authors [96] showed that a FoPL can improve the accuracy of judgments about the nutritional quality of foods and beverages, and consumers are encouraged to make healthier choices if nutritional criteria are present in a clear way. Consumers, over time, have become increasingly able to distinguish the various degrees of the healthiness of foods through the various types of labels and key nutrient information [98]. They inform the consumer toward healthier choices [97]. Grunert et al. [94] added that nutrition labels do not increase the healthy choices made by consumers, but they increase the ability of consumers to make informed choices and understand which products are healthier. According to Talati et al. [99,100], consumers think that FoPL are valuable sources of nutrition information since they are controlled by regulators and that health claims should be reliable, relevant, and informative, while FoPL should be trustworthy and easy to understand. However, there is still a need to implement a uniform, consistent and credible labelling system [101].

Among the proposed FoPL, in this timeframe, the SM label has been introduced. Roberto et al. [102] affirmed that the label was able to increase consumers’ ability to understand the calories per serving of food while decreasing their ability to decide the adequate quantity of products to consume. Additionally, he argued that Smart Choices and Multiple Traffic Light (MTL) perform similarly in educating the consumer, while MTL labels are better in estimating nutrient amounts since the SM does not highlight a detailed depiction of nutritive values [85]. In general, the MTL label seems to be the most effective FoPL [103]. By examining the effects of saturated fat and sodium on consumer health, consumers tended to respond more favorably to the numbers rather than percentages [104]. Moreover, MTL can rely both on colours and text, which are relevant elements in the labelling system, as they help draw attention to the label and consequently to its contents [96]. Research carried out by Wąsowicz et al. [103] showed that Multiple Traffic Light (MTL) colours, such as green, amber and red, were associated with healthy fruits and vegetables, and their combination was evaluated as appropriate to highlight unhealthy products. Similarly, the Health Logo (HL) was associated with a sense of healthiness [105].

Another analysed FoPL is the organic label. In fact, food companies that have used the organic label in their products have definitely obtained positive results because of healthier associations attached to foods with the presence of organic cues [106].

In addition, FoPL are starting to appear on menus, where, primarily, calorie information should be presented. One idea is to base menu labelling on the colour scheme used in the “traffic light” labelling system, where different colours indicate levels of various elements and can even educate consumers [101,107]. The introduction of menu labelling has led to different consequences. For example, in fast-food restaurants, there was no obvious alteration in the products purchased by consumers. On the other hand, this alteration was most apparent in settings such as miscellaneous and service restaurants, where customers were encouraged to purchase less energy [108].

The networks in this period welcomed the new regulations developing a sequence of studies to test the effects of the labels on consumers. Oftentimes, they tended to compare the alternatives in different countries without arriving at univocal results.

##### Period: 2017–2022—Opening up to alternatives and challenging the mainstream

In the third period, from 2017 to the present, important new research streams emerged. This period is characterized by the introduction of the NS in France (2017) and then over time in other countries, such as Belgium (2018), Germany and Switzerland (2020) and Luxembourg (2021) (Table 12 and Table 13). In this context, the two co-citation networks of the previous period continue to develop new studies along two trajectories: one group focused on the NS (co-citation network 3 in blue) and a second focused on the other labels (co-citation network 4 in red). In co-citation network 3, the strongest links are those between Chantal and Julia (same author, with two different references), Julia and Egnell, Julia and Donnenfeld, Chantal and Ducrot, Vith and Ducrot, Ducrot and Crosetto and Crosetto and Hersey, with a strong recurrence of the same country of affiliation. As for co-citation network 4, the strongest links are those between Grunert and Graham. In addition, there emerged another co-citation network (5, in green) focused on the importance of the FoPL and the relevance of the chosen labels for policymakers. In this network, there is an equally distributed weight across all the links.

#### 3.2.5. Co-Citation Network 5

On 16 December 2016, the obligation to provide nutrition information began, as regulated by Regulation (EU) 1169/2011 [17]. Since then, there has been a consolidation of past research streams focused on specific FoPL and the formation of new ones (Table 14).

The current co-citation network evolved from the previous period, and the focus continues to be on the NS, which was officially adopted in France in 2017 and was subsequently adopted by several countries. The network includes new authors [24,108,109,110] and new contributions from authors already present in the past period, such as Julia [111,112,113].

One of the first contributions in this timespan is from Julia et al. [111]. In their research, the authors state that the NS is a valid FoPL for increasing the chance of making salutary choices [111]. Egnell et al. [24] and Sarda et al. [114] added that the NS outperforms other summary graded formats, showing that it can rely on higher objective understanding [24]. The NS label is one of the most simplified labels conveying to consumers a clearer and easier message [25]. Similarly, the study conducted by Talati et al. [25] states that the NS is the most effective FoPL in terms of consumer understanding. In addition, Egnell et al. [24] demonstrated that FoPL nutrition labels had an important influence on the understanding of the healthiness of the products, and the NS led to a greater objective understanding relative to FoPL. While the study conducted by Crosetto et al. [110] has demonstrated that a simpler food label performs better than an analytical and detailed food label. Julia et al. [115] added that the NS, at this time, shows an increase in awareness due to communication and its increasing presence in supermarkets and online platforms.

The study conducted by Egnell et al. [31,108] also confirms that the NS label, due to its summarized and graduated colour format, is more effective than the label in encouraging students, the subjects of the research under review, to shop for food of higher nutritional quality.

Additionally, Egnell et al. [116] showed that the summarized and graded format of the NS is favorably perceived and understood, regardless of the type of consumer.

#### 3.2.6. Co-Citation Network 6

Similarly, over this period of time, the European Union, within the recent “Farm-to-Fork” strategy, initiated an attempt to harmonize FoPLs, while, at the same time, scholars started integrating the concept of FoPL harmonization in their work [20]. In the last regulatory attempt, the EU, with the “Inception Impact Assessment”, focused on finding a unifying solution by assessing the differential performance of FoPL, such as the NS (already adopted in different countries), the NutrInform Battery (proposed by Italy), and the Keyhole (utilized by Sweden and other Scandinavian countries). As a consequence, several authors continued to conduct research about the other label and on labels’ understanding. More precisely, Meijer [117], Findling et al. [118], Andrews [119], and Burton et al. [120], in this last period, carried out new research and added new contributions to this co-citation network.

Some relevant advancements have been proposed by Findling et al. [118], who, drawing on studies of Roberto et al. [107,121], stated that the presence of the FoPL definitely improved consumers’ nutritional knowledge significantly. A new contribution was added by Rybak et al. [122], which showed that the perception of the healthiness of products is influenced by their nutritional characteristics and the clarity of labels. The research conducted by Andrews et al. [119] seeks to understand which labels are most beneficial and, therefore, most effective. In this context, Stop Sign labels appear to be most effective in helping nutrient ratings, disease risk perceptions, brand attitudes and purchase intentions by targeting high levels of negative nutrients (saturated fat, sodium).

On the other hand, the same research states that the situation changes when looking at nutrient accuracy scores for a wider range of six nutrients. In this case, the study finds Multiple Traffic Light (MTL) labels to be more effective [119].

Similarly, the study conducted by Findling et al. [118] identified that different label preferences are noted in different situations. In the same study, the MTL allowed to better estimate the levels of nutrients than all other labels [118].

Zhang et al. [123] confirmed another very important issue regarding the choice of products according to demographic factors. In fact, this study confirmed what has been said in previous studies: that different demographic characteristics lead to different uses of nutrition labels. For example, the current study shows that women pay much more attention to information on labels.

Considering that within 2022, the EU plans to define a final set of regulations to ensure standardized front-of-pack nutrition labelling in Europe, strong competition among the different proposals arose due to their ability to promote the consumption of some products while discouraging others. Indeed, it has been one of the main issues highlighted by local authorities in some countries to indicate how the implementation of some FoPL would drastically penalize the consumption of certain categories of products with serious effects on the industry. Consequently, some streams of research consolidated their position and others, such as MTL, drastically reduced the scenario after the Brexit.

#### 3.2.7. Co-Citation Network 7

In this last period, a fifth co-citation network emerged. In this co-citation network, the most frequent topics analysed are related to the study of other labels, although the authors also make comparisons with NS. In addition, the issue of the importance of the use of FoPL as well as the relevance for policymakers of which labels to choose emerged. There came a critical time for governments; different situations emerged, and there were historical events that lead to changes. This situation brought about a decrease in the relevance of the NS, which had become one of the central labels.

The study conducted by Talati et al. [124] highlighted that FoPL may, even unconsciously, improve the quality of the chosen product. Another study, conducted by Talati et al. [125], tested consumer awareness of how it changes if the FoPL label is present. The study confirmed the importance of FoPL, stating that consumers are more aware of healthier choices when FoPL labels are displayed [124].

Pettigrew et al. [126] confirmed what Talati et al. [124,125] stated, asserting that interpretative FoPL labels help consumers to make healthier food choices more than non-interpretive ones (RIs) [126,127]. Therefore, Pettigrew et al. [126] even confirmed previous studies that FoPL improves consumer awareness. Nevertheless, the performance of different labels changes depending on various factors, and color-coded information with a summarizing graduated graphic design is the most effective element of the label. The study, conducted by Kelly et al. [128], echoes the importance of product information and shredded light on the acceptance of FoPL.

In addition, Egnell et al. [116] compared all types of FoPL, including the NS. The result of this study states that among all the different FoPL, there are no significant differences affecting food choices and perceptions. Yet, the NS shows the best result in terms of ranking products according to nutritional quality [126].

In the same year, Fialon et al. [129] confirmed that the presence of FoPL improved the perception of nutritional quality and confirmed that the NS is the best FoPL to highlight the nutritional quality of food.

### 3.3. Network Analysis

The analysis of network measures and community-driven measures highlights a positive correlation between the betweenness centrality, closeness centrality, PageRank and H-index, indicating that those more central in the network have a higher likelihood of influencing other authors. As shown in Table 4, the H-index is positively correlated to closeness centrality. This suggests that users with a shorter average distance to all other vertices in the network (i.e., close to the other nodes) are more influential and impactful. Similarly, those authors connected to the highest number of the shortest paths between two vertices are those who are more likely to control communication since several links are passing through those paths [130]. As regards the PageRank index [59], the most-connected authors (i.e., relevant nodes) are those with a higher H-index (Table 15).

In line with correlation analysis, Table 16 describes that, on average, the authors with the highest H-Index are those with higher levels of betweenness and PageRank. As a result, network centrality mirrors the H-index, showing authors that gained a central position in their specific sub-networks. However, with the literature being a ground for comparison, a higher centrality in the network does not imply incontrovertible findings.

The results highlight how some authors are more linked than others and contribute, in terms of articles produced, to the same topics or fields, reinforcing mutual strength in the network, despite the variety of topics discussed. There is a high level of citations for network 1, which reflects the first contributions to the topic. These are naturally cited as foundation articles. Similar results are seen for co-citation network 3, which mainly reflects the contributions to the NS (Table 17).

Relevant results indicate the need to further investigate the structure of the literature and understand whether relevant gaps have been overlooked over time. This network density might produce over-concentration of attention on mainstream topics, despite the presence of other alternative, less convergent streams of equal theoretical validity. The risk for policy-makers when assessing FoPL by leveraging only a quantitative output-based approach is to overweight the implication merely deriving from the convergence on a topic and to ignore underweighted results in the co-citation networks. However, the goal of the literature is to compare, contrast and upgrade scientific evidence through the comparison of different streams, especially when a multifaceted nature of perspectives is present and an overall convergence on a sole topic does not exist.

In the second part of the analysis, to further assess the evolution of the networks and fulfil the existing gaps, we qualitatively analysed and discussed the topics covered in the networks and the positions of the authors within them.

#### What’s Next with the Most Recent Contributions?

**Figure 8:**
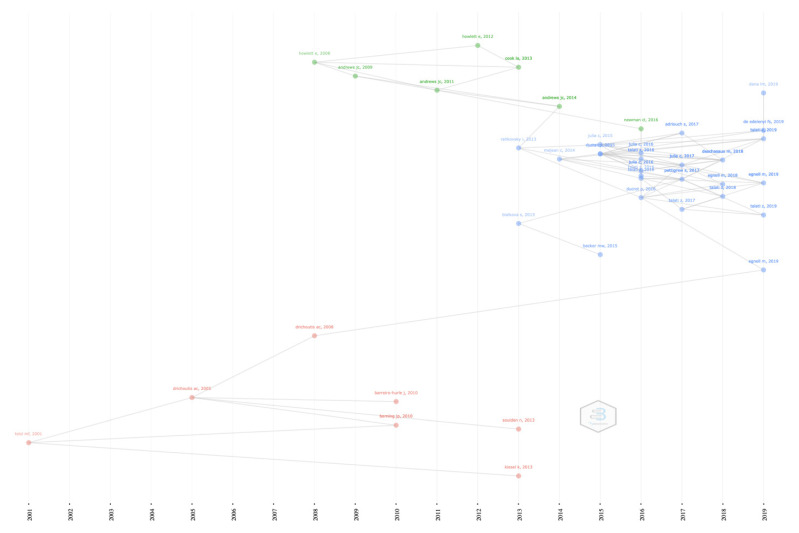
Co-citation evolution over time.

The NS-oriented co-citation network mainly evolved in the last two decades, becoming more and more central. Conversely, co-citation network 2 significantly reduced its co-citations, whereas Co-citation network 3 partially increased its co-citations. Co-citation network 3 did not exist in the 1989–2011 timespan but drastically increased the share of co-citations during the 2012–2016 period and consolidated it in the last analysed period (2017–2022). The other co-citation networks have lost several ties, indicating the lack of temporariness of the links (Figure 8).

This stage of the research showed that relevant components exist and evolve within the broader network over time. Authors within the main network are englobed in co-citation subgroups, sometimes driven by similar countries of affiliation, that evolved in the three analysed periods. Some co-citation subgroups did not show strong ties, and the members disappeared from the network due to a reduced number of articles produced in the analysed timespan or tended to be absorbed by more consolidated subgroups. The NS-oriented co-citation network firstly appeared in the 2011–2016 timespan and consolidated itself during the 2017-2022 period, increasing the number of published articles, the number of co-citations and the number of studies about the NS.

More recent contributions continue to highlight the relevance of FoPL and their positive effects on consumers’ choices [5,131,132,133,134,135,136,137]. However, there is still a multitude of valid effects demonstrated without a full convergence of the authors [3].

New studies on FoPL, reveal through online experiments that NS could be a FoPL implemented worldwide [117]. Andreeva et al. [138] highlight several studies pointed out the superiority specifically of NS, followed by Multiple Traffic Lights and Simplified Food Labelling System. On the other hand, studies which criticize the NS have emerged, particularly regarding the absence of vitamins/minerals and sustainability/environmental impact measures from the scoring algorithm, the absence of information on additives or pesticides in food [106]. In addition, Touvier et al. [139] affirms that the NS does not present any conflict of interest, and enlighten the quantum of scientific evidence to demonstrate independence, effectiveness, and usefulness for consumers and public health. A further implication is included, taking its ability to outperform on some dimension other existing labels and linking to the hypothesis that other labels are supported by industry lobbying groups [139].

Different results than the previous ones emerged from a study conducted by Oswald et al. [140] that compared two different FoPLs, one colour-coded and one not, and found little effect on consumers. In fact, the study states that consumers likely see the label information as additional and supplemental to their choice and believe more educational campaigns are needed to increase understanding. Finally, the study does not arrive at one label being uniquely and consistently all the important dimensions better than another.

More recent evidence, highlighted the greater effectiveness of colour-coded labels (such as Nutri-Score and Multiple Traffic Light) over the Reference Intakes, Warning Label and Health Star Rating. The labels continue to perform better in one dimension of decision making—the objective understanding—in 18 countries [141], in Netherlands [142], French adolescents [143], Chile [144] and, other studies, confirm that is associated to a reduction of consumed harmful products [144]. Conversely, other research discussed the effectiveness of bundled FoPL composed by Health Star Rating and Warning Labels [145] in a real-world selection task. As for Warning Labels, other authors [146] showed the higher utility attached by consumers that is associated to the willingness to pay higher prices for products with such labels. The efficacy of bundled FoPL is also confirmed in another study of Touvier et al. [147] in which combined FoPL and ratings are described as more beneficial for consumers.

In the meanwhile, the benefits of front-of-pack labels have also been outlined in Southeast Asia [148], Ireland, the US [149,150], Poland [151], Portugal [2] and Mexico [152]. In other countries, it has been argued that there is a reactance to the acceptance of the Nutri-Score [134]. In Italy, for instance, Fialon et al. [153] argued that policy-makers and industry-expert oppose the Nutri-Score with a narrative based on the protection of the Made-in-Italy products and the Mediterranean Diet. Other recent research contends that the Nutri-Score is the most attacked label for economic or ideological interests [154] and is involved in an unfair tug-of-war even if constantly associated with high effectiveness in several studies and settings [133,155]. The Nutri-Score label is also associated with several comments’ letters produced in response to research evidence and articles to protect its superiority [136,138,156,157,158,159,160], as well as to higher levels of online citations [161].

All valid results must be confronted with other valid evidence that proves, in 2022, the superiority of other FoPL, such as the NutrInform Battery [162,163], Multiple Traffic Light [164,165,166,167,168], Warning Labels [169,170,171], the KeyHole Logo [5], Health Star Ratings [172] or suggest an improvement of the Nutri-Score [172]. All the aforementioned studies highlight higher performances of the cited FoPL, mainly in comparative studies, confirming the non-convergence of results when assessed through different relevant parameters in consumers’ decision-making.

This evidence equally contributes to those European Union principles which demand strong science to prove safety [173,174] and to the debate on FoPL. In addition, even after the coronavirus pandemic, an increasing need to regulate the food industry through the adoption of a univocal FoPL has been registered. The main concern raised about the final European decision might be how to compare all these valid results and find the univocal FoPL in the short run.

## 4. Discussion

To the extent of our knowledge, this research is the first bibliometric analysis corroborated by a systematic literature review on the increasingly relevant topic of FoPL over a time period ranging from 1989 to 2022 and on 170 articles. On top of this amount, we dedicated a specific section to 49 articles published in the last months for a total of 219 articles. The evidence arising from the analysis mainly refers to the evolution of policy-making initiatives, FoPL theory and their interrelations.

The regulation flow evolved in the last 33 years, observing the consumer with different lenses. In 1990, the Council Directive 90/496/EEC [13] was issued, respecting a paradigm that considers the information able to guide individuals in their rational decision-making process. During this period, the nutrition labelling systems are considered items to support a decision and not to nudge it toward healthier choices [14]. Contemporarily, the literature has studied the effects of the nutrition labelling systems and FoPL, mainly arriving at non-univocal results and suggesting relevant implications to improve the effectiveness of this tool.

Indeed, in 2008, with the new Proposal, the EU Commission suggested new implementations for FoPL which are able to orientate consumers’ decisions. A shift in the paradigm mainly arose from the increasing level of obesity in Europe and the recognition of the bounded rationality of individuals and factors affecting the decisions (Simon, 1954).

A new way to conceive FoPL and their roles in the consumer decision-making process led to the Regulation (EU) 1169/2011 [17] and the mandatory application of nutrition information on packaging. In this evolutionary stage, regulators wanted to make FoPL mandatory and, in addition, easy to understand for the average consumer. In contraposition with the “information paradigm”, the Regulation (EU) 1169/2011 laid down the ground for strong competition among the different proposals in the EU.

Since the obligations provided by regulation 1169/2011 became mandatory in 2016, some European proposals started to form their contributions beforehand and consolidated their position in the subsequent years. In fact, the period between 2011 and 2022 welcomed new FoPL proposed by Member States, and several studies about these new concepts appeared even before their launch in the market. For instance, studies on NS mainly appeared beginning in 2013, and the FoPL was launched in 2017.

In this test phase, the literature assessed the effectiveness and the effects on new proposals but, just as the previous timespan, mainly arrived at opposite results. In this uncertain context, the main difference able to characterize the strength of the studies is the existence of a network to support the evidence. However, although some networks appear more consolidated in the literature, as for the co-citation network 3, which focused on NS, and others that were more fragmented, policy-makers should clearly recognize that the degree of support toward evidence does not imply the non-validity of other—equally important—evidence.

This appears confirmed by the plethora of studies indicating different performance for subjective understanding [22,32,33], objective understanding [23,24,116] and acceptance [37]. Part of the previous literature utilized the conceptual framework developed by Grunert and Wills [26], focusing on consumers’ objective and subjective understanding and leading to controvertible evidence [22,23,25,27,30,31,32,33,109] of the absolute superiority of a specific FoPL capable of affecting consumer behaviour toward healthier lifestyles.

However, in the periods 2012–2016 and 2017–2022, only one co-citation network appears, indicating a reinforced convergence across the topics discussed. In this stream, the authors mainly provide evidence of the results of the NS label and its different effects on affecting consumers’ choices. In this coherent evolution, the network increased the level of internal citations, even reinforcing the number of articles produced over the years. The majority of them, as exposed before, converge on the same topic and form a separate stream of research in relation to the others. Moreover, as seen through the correlation analysis, by increasing the number of co-citations, there is a back-forward propagation effect that lead articles to increase their centrality in the network.

Nevertheless, more than half of the literature appears oriented toward other results (see Table 7, Table 8, Table 10, Table 18 and Table 19), even if they are more fragmented and not consolidated in a common stream of research. The other co-citation networks temporarily converge on the same topics or findings within the limits of the analysed timeframe and tend to completely disappear or redistribute in the other networks along the entire time horizon. These other components of the literature mainly investigate other label types and their effects. Other investigations, as for the co-citation networks 1 and 4, mainly focused on understanding the factors that drive the comprehension of FoPL and the differences related to socio-demographic characteristics. On the other hand, the co-citation networks 2 and 3 are more oriented towards the antecedent of the usage of FoPL. Similarly, additional contributions dared to explore untapped avenues, created the basis for alternative frameworks or point of view, developing alternative and complementary conceptual frameworks to help consumers’ decision-making toward utilization and acceptance of FoPLs that support informed decisions toward healthier diets [33].

However, there are still many under-researched areas, such as those who behaviourally assess the acceptance of FoPL and their real usage rather than the perceptual use. In this perspective, there is a need to open the ground to new research and evidence for supporting a FoPL that will lead consumers to improve food choices. Similarly, future studies could both focus on the role of trust towards algorithmic-based FoPL and the differences that result when various labels are compared in the context of disclosure of the computational methods. Moreover, in the current debate at the European level on what would be the best FoPL to support customers’ choices, limited effort has been dedicated to analysing the potential effects of bundling existing FoPL. Additionally, it might also be interesting to test whether relevant differences in terms of usefulness and ease of use occur when comparing different FoPLs. From another perspective, scholars could assess the role of moderators, such as the trust, the level of experience of the users, the type of users and the type of usage.

## 5. Conclusions

The most recent goal of EU policy-makers is to find a harmonized and universal labelling system to adapt in all European countries. However, observing the structure of the extant literature, there might be two current risks that should be avoided. The first risk is to outline a labelling scheme that is not fully supported by converging evidence as derived from multiple different constructs. The second one refers to the risk of implementing a labelling scheme grounded on valid results and high levels of citations, supported by a network of authors, but overlooking the fragmentation of other valid positions in the literature that together contribute to depicting an environment in which the different and still valid results reflect the diversity of alternatives that are equally effective, but less supported. In conclusion, the right choice of FoPL would benefit both consumers and the food industry, but there are still additional knowledge and usage gaps that must be fulfilled to define the proper universal option that supports consumers toward healthier and more informed food choices.

## Figures and Tables

**Table 1:** Adapted EU Taxonomy and an example of front-of-pack labels (EU Commission, 2021).

Taxonomies		Examples
**Nutrient-Specific Labels**: FoPL * that provide detailed information about certain nutrients (fat, saturates, sugars, salt, and energy value) with an objective description of the quantities contained in the food	**Numerical Labels**: non-interpretative (non-evaluative) labels that provide numerical information on the content of four nutrients (fat, saturates, sugars, and salt) and on the energy value, as well as on how much this represents as a percentage of the daily reference intake	**NutrInform Battery** (hereinafter as “NiB”) 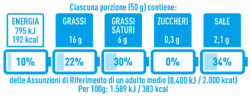
	**Colour-Coded Labels**: labels providing numerical information on the content of four nutrients (fat, saturates, sugars, salt) and on the energy value, as well as on how much this represents as a percentage of the daily reference intake. Colours are used to classify those nutrients as “low” (green), “medium” (amber) or “high” (red)	**Multiple Traffic Light** (hereinafter as “MTL”) 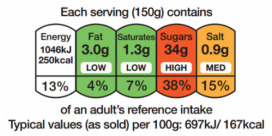
**Summary Labels**: FoPL * that provide a synthetic assessment of the product’s overall nutritional healthfulness that is sometimes the result of an algorithmic computation	**Endorsement Logos**: labels providing a synthetic appreciation of a product’s overall nutritional value through a positive (endorsement) logo that is applied only to foods that comply with nutritional criteria	**Keyhole logo** 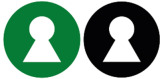
	**Graded Indicators**: labels providing a synthetic appreciation of a product’s overall nutritional value through a “graded indicator” that provides graded information on the nutritional quality of foods and is applied to all food products	**Nutri-Score** (hereinafter as “NS”) 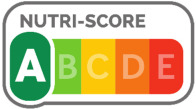

* FoPL: Front-of-Pack labels.

**Table 2:** Most-cited articles.

Article	DOI	Total Citations	Average Citation per Year
DRICHOUTIS AC, 2005, EUR REV AGRIC ECON	10.1093/erae/jbi003	177	9.83
ANDREWS JC, 2011, J PUBLIC POLICY MARK	10.1509/jppm.30.2.175	121	10.08
BARREIRO-HURLE J, 2010, FOOD POLICY	10.1016/j.foodpol.2009.12.006	119	9.15
ANSELMSSON J, 2014, J PROD BRAND MANAG	10.1108/JPBM-10-2013-0414	85	9.44
DUCROT P, 2016, AM J PREV MED	10.1016/j.amepre.2015.10.020	84	12.00
ANDREWS JC, 2009, J PUBLIC POLICY MARK	10.1509/jppm.28.1.41	79	5.64
TEISL MF, 2001, AM J AGR ECON	10.1111/0002-9092.00142	79	3.59
IKONEN I, 2020, J ACAD MARK SCI	10.1007/s11747-019-00663-9	62	20.67
KIESEL K, 2013, INT J IND ORGAN	10.1016/j.ijindorg.2010.11.002	62	6.20
BECKER MW, 2015, FOOD POLICY	10.1016/j.foodpol.2015.08.001	57	7.13
HOWLETT E, 2008, J PUBLIC POLICY MARK	10.1509/jppm.27.1.83	57	3.80
BIALKOVA S, 2013, FOOD POLICY	10.1016/j.foodpol.2013.04.010	56	5.60
NEWMAN CL, 2014, J RETAIL	10.1016/j.jretai.2013.11.001	53	5.89
DUCROT P, 2015, PLOS ONE	10.1371/journal.pone.0140898	51	6.38
ELLIOTT CD, 2009, CAN J COMMUN	10.22230/cjc.2009v34n3a2220	50	3.57
TALATI Z, 2016, NUTRIENTS	10.3390/nu8120787	50	7.14
OZRETIC-DOSEN D, 2007, J BUS RES	10.1016/j.jbusres.2006.10.011	49	3.06
DRICHOUTIS AC, 2008, EUR J HEALTH ECON	10.1007/s10198-007-0077-y	48	3.20
RAHKOVSKY I, 2013, FOOD POLICY	10.1016/j.foodpol.2013.08.013	47	4.70
BARREIRO-HURLE J, 2010, J AGRIC ECON	10.1111/j.1477-9552.2010.00247.x	44	3.39

**Table 3:** Most prolific countries and the number of citations.

Country	Frequency	Total Citations
USA	194	1115
France	155	658
Australia	127	371
UK	31	43
Spain	28	163
Germany	21	54
Canada	20	132
Italy	20	45
China	17	50
Netherlands	12	118
Greece	11	225
Sweden	9	85
Switzerland	9	90
Belgium	8	24
Brazil	7	1
Denmark	7	0
Poland	7	0
India	6	0
Finland	5	4
Mexico	4	0

**Table 4:** Composition of co-citation network 1 in terms of authors, articles, year, country of authors and citation.

	Id	Authors	Articles	Year	Country of Authors	Citation
1989–2011	1	Burton, S., Biswas, A., & Netemeyer, R.	Effects of Alternative Nutrition Label Formats and Nutrition Reference Information on Consumer Perceptions, Comprehension, and Product Evaluations	1994	USA	139
	2	Burton, S., & Andrews, J. C.	Age, Product Nutrition, and Label Format Effects on Consumer Perceptions and Product Evaluations	1996	USA	146
	3	Keller, S. B., Landry, M., Olson, J., Velliquette, A. M., Burton, S., & Andrews, J. C.	The Effects of Nutrition Package Claims, Nutrition Facts Panels, and Motivation to Process Nutrition Information on Consumer Product Evaluations	1997	USA	282
	4	Andrews, J. C., Netemeyer, R. G., & Burton, S.	Consumer Generalization of Nutrient Content Claims in Advertising	1998	USA	421
	5	Burton, S., Garretson, J. A., & Velliquette, A. M.	Implications of Accurate Usage of Nutrition Facts Panel Information for Food Product Evaluations and Purchase Intentions	1998	USA	184
	6	Mitra, A., Hastak, M., Ford, G. T., & Ringold, D. J.	Can the Educationally Disadvantaged Interpret the FDA-Mandated Nutrition Facts Panel in the Presence of an Implied Health Claim?	1999	USA	121
	7	Roe, B., Levy, A. S., & Derby, B. M.	The Impact of Health Claims on Consumer Search and Product Evaluation Outcomes: Results from FDA Experimental Data	1999	USA	594
	8	Andrews, J. C., Burton, S., & Netemeyer, R. G.	Are Some Comparative Nutrition Claims Misleading? The Role of Nutrition Knowledge, Ad Claim Type and Disclosure Conditions	2000	USA	272
	9	Grunert, K. G.	Current issues in the understanding of consumer food choice	2002	Denmark	655
	10	Kozup, J. C., Creyer, E. H., & Burton, S.	Making Healthful Food Choices: The Influence of Health Claims and Nutrition Information on Consumers’ Evaluations of Packaged Food Products and Restaurant Menu Items	2003	USA	863
	11	Roe, B., & Sheldon, I.	Credence good labeling: The efficiency and distributional implications of several policy approaches	2007	USA	246
	12	Grunert, K. G., & Wills, J. M.	A review of European research on consumer response to nutrition information on food labels	2007	Denmark, Belgium	1341
	13	Grunert, K. G., Fernández-Celemín, L., Wills, J. M., Storcksdieck genannt Bonsmann, S., & Nureeva, L.	Use and understanding of nutrition information on food labels in six European countries	2007	Denmark, Belgium	440
	14	Howlett, E., Burton, S., & Kozup, J.	How Modification of the Nutrition Facts Panel Influences Consumers at Risk for Heart Disease: The Case of Trans Fat	2008	USA	94
	15	Teisl, M. F., Radas, S., & Roe, B.	Struggles in optimal labelling: how different consumers react to various labels for genetically modified foods	2008	USA	27
	16	Feunekes, G. I., Gortemaker, I. A., Willems, A. A., Lion, R., & Van Den Kommer, M.	Front-of-pack nutrition labelling: Testing effectiveness of different nutrition labelling formats front-of-pack in four European countries	2008	The Netherlands	558
	17	Vyth, E. L., Steenhuis, I. H., Mallant, S. F., Mol, Z. L., Brug, J., Temminghoff, M., ... & Seidell, J. C.	A Front-of-Pack Nutrition Logo: A Quantitative and Qualitative Process Evaluation in the Netherlands	2009	The Netherlands	132
	18	Burton, S., Howlett, E., & Tangari, A. H.	Food for Thought: How Will the Nutrition Labeling of Quick Service Restaurant Menu Items Influence Consumers’ Product Evaluations, Purchase Intentions, and Choices?	2009	USA	204
	19	Wills, J. M., Grunert, K. G., Celemin, L. F., & BoNSMANN, S. S. G.	Do European consumers use nutrition labels	2009	Denmark, Belgium	12
	20	genannt Bonsmann, S. S., & Grunert, E. K. G.	Food Labelling to Advance Better Education for Life	2010	Denmark, Belgium	57
	21	Grunert, K. G., Wills, J. M., & Fernández-Celemín, L.	Nutrition knowledge, and use and understanding of nutrition information on food labels among consumers in the UK	2010	Denmark, Belgium	712
	22	Andrews, J. C., Burton, S., & Kees, J.	Is Simpler Always Better? Consumer Evaluations of Front-of-Package Nutrition Symbols	2011	USA	229

**Table 5:** The relative strength of the nodes within the co-citation network.

Node	Co-Citation Network	Betweenness	Closeness	PageRank
Burton S.	1	475.7513	0.0052	0.0267
Kozup J.C.	1	38.6570	0.0045	0.0299
Grunert K.G.	1	34.7742	0.0044	0.0212
Roe B.	1	15.5871	0.0044	0.0263
Keller S.B.	1	15.5860	0.0044	0.0282
Mitra A.	1	7.0525	0.0042	0.0250
Moorman C.	1	5.9589	0.0041	0.0265
Garretson J.A.	1	4.0021	0.0042	0.0202
Balasubramanian S.K.	1	3.2255	0.0043	0.0252
Feunekes G.I.	1	2.3474	0.0042	0.0186
Jacoby J.	1	1.2850	0.0042	0.0195
Chandon P.	1	0.9064	0.0040	0.0192
Chaiken S.	1	0.4333	0.0028	0.0234
Cacioppo J.T.	1	0.4333	0.0028	0.0234
Andrews J.C.	1	0.0000	0.0027	0.0196

**Table 6:** Composition of co-citation network 2 in terms of authors, articles, year, country of authors and citation.

Year	#	Authors	Articles	Year	Country of Authors	Citation
1989–2011	1	Schucker, R. E., Levy, A. S., Tenney, J. E., & Mathews, O.	Nutrition Shelf-Labeling and Consumer Purchase Behavior	1992	USA	66
	2	Ippolito, P. M., & Mathios, A. D	New Food Labeling Regulations and the Flow of Nutrition Information to Consumers	1993	USA	103
	3	Wang, G., Fletcher, S. M., & Carley, D. H.	Consumer Utilization of Food Labeling as a Source of Nutrition Information	1995	USA	142
	4	Guthrie, J. F., Fox, J. J., Cleveland, L. E., & Welsh, S.	Who Uses Nutrition Labeling, and What Effects Does Label Use Have on Diet Quality?	1995	USA	378
	5	Levy, A. S., Fein, S. B., & Schucker, R. E.	Performance Characteristics of Seven Nutrition Label Formats	1996	USA	191
	6	Levy, A. S. & Fein, S. B.	Consumers’ Ability to Perform Tasks Using Nutrition Labels	1998	USA	194
	7	Teisl, M. F., Levy, A. S., & Derby, B. M.	The Effects of Education and Information Source on Consumer Awareness of Diet-Disease Relationships	1999	USA	66
	8	Coulson, N. S.	An application of the stages of change model to consumer use of food labels	2000	UK	162
	9	Teisl, M. F., Bockstael, N. E., & Levy, A.	Measuring the Welfare Effects of Nutrition Information	2001	USA	237
	10	Wansink, B., Painter, J., & Ittersum, K. V.	How Descriptive Menu Labels Influence Attitudes and Repatronage	2002	USA	11
	11	Wansink, B., Sonka, S. T., & Hasler, C. M.	Front-label health claims: when less is more	2004	USA	196
	12	Wansink, B., Painter, J., & Ittersum, K. V.	How Diet and Health Labels Influence Taste and Satiation	2004	USA	105
	13	Drichoutis, A. C., Lazaridis, P., & Nayga Jr, R. M.	Nutrition knowledge and consumer use of nutritional food labels	2005	Greece	425
	14	Wansink, B., & Chandon, P.	Can “Low-Fat” Nutrition Labels Lead to Obesity?	2006	France	919
	15	Loureiro, M. L., Gracia, A., & Nayga Jr, R. M.	Do consumers value nutritional labels?	2006	Spain	124
	16	Drichoutis, A. C., Lazaridis, P., & Nayga Jr, R. M.	An assessment of product class involvement in food-purchasing behavior	2007	Greece, USA	91
	17	Roe, B. & Teisl, M. F.	Genetically modified food labeling: The impacts of message and messenger on consumer perceptions of labels and products	2007	USA	130
	18	Gracia, A., Loureiro, M. L., & Nayga Jr, R. M.	Do consumers perceive benefits from the implementation of a EU mandatory nutritional labelling program?	2007	Spain	107
	19	Drichoutis, A. C., Lazaridis, P., Nayga, R. M., Kapsokefalou, M., & Chryssochoidis, G.	A theoretical and empirical investigation of nutritional label use	2008	Greece, USA	133
	20	Radas, S., Teisl, M. F., & Roe, B.	An Open Mind Wants More: Opinion Strength and the Desire for Genetically Modified Food Labeling Policy	2008	Croatia, USA	35
	21	Variyam, J. N.	Do nutrition labels improve dietary outcomes?	2008	USA	241
	22	Golan, E., & Unnevehr, L.	Food product composition, consumer health, and public policy: Introduction and overview of special section	2008	USA	78
	23	Teisl, M. F., Radas, S., & Roe, B.	Struggles in optimal labelling: how different consumers react to various labels for genetically modified foods	2008	USA, Croatia	27
	24	Drichoutis, A. C., Nayga, Jr, R. M., & Lazaridis, P.	Can Nutritional Label Use Influence Body Weight Outcomes?	2009	Greece, USA	68
	25	Gracia, A., Loureiro, M. L., & Nayga Jr, R. M.	Consumers’ valuation of nutritional information: A choice experiment study	2009	Spain, USA	137
	26	Puduri, V., Govindasamy, R., & Onyango, B.	Country of origin labelling of fresh produce: a consumer preference analysis	2009	USA	22
	27	Barreiro-Hurlé, J., Gracia, A., & De-Magistris, T.	Market implications of new regulations: impact of health and nutrition information on consumer choice	2009	Spain	29
	28	Drichoutis, A. C., Lazaridis, P., & Nayga Jr, R. M.	ON CONSUMERS’ VALUATION OF NUTRITION INFORMATION	2009	Greece, USA	45
	29	Drichoutis, A. C., Lazaridis, P., & Nayga Jr, R. M.	Would Consumers Value Food-Away-From-Home Products With Nutritional Labels?	2009	Greece	42
	30	Barreiro-Hurlé, J., Gracia, A., & De-Magistris, T.	Does nutrition information on food products lead to healthier food choices?	2010	Spain	269
	31	Barreiro-Hurlé, J., Gracia, A., & De-Magistris, T.	The Effects of Multiple Health and Nutrition Labels on Consumer Food Choices	2010	Spain	91
	32	Golan, E., & Kuchler, F.	The Effect of GM Labeling Regime on Market Outcomes	2011	USA	6

**Table 7:** The relative strength of the nodes within co-citation network 2.

Node	Co-Citation Network	Betweenness	Closeness	PageRank
Nayga R.M.	2	163.0369	0.0048	0.0336
Wansink B.	2	127.1826	0.0047	0.0257
Stigler G.J.	2	80.0827	0.0045	0.0336
Ippolito P.M.	2	53.3569	0.0047	0.0235
Variyam J.N.	2	28.6655	0.0045	0.0215
Szykman L.R.	2	27.1180	0.0046	0.0210
Levy A.S.	2	18.1432	0.0045	0.0193
Kim S.Y.	2	6.7404	0,0045	0.0256
Nayga R. M. Jr.	2	6.7404	0.0045	0.0256
Wang G.J.	2	6.7404	0.0045	0.0256
Drichoutis A.C.	2	3.6414	0.0044	0.0240
Govindasamy R.	2	3.2936	0.0044	0.0253
Guthrie J.F.	2	1.0827	0.0044	0.0205
Kim S.Y.	2	1.0827	0.0044	0.0205
Mcleanmeyinsse P.E.	2	1.0827	0.0044	0.0205
Gracia A.	2	1.0537	0.0044	0.0209
Coulson N.S.	2	0.9562	0.0044	0.0231
Caswell J.A.	2	0.0000	0.0024	0.0157
Parmenter K.	2	0.0000	0.0027	0.0207
Teisl M.F.	2	0.0000	0.0026	0.0186
Blaylock J.	2	0.0000	0.0027	0.0207
Golan E.	2	0.0000	0.0043	0.0190
Grossman M.	2	0.0000	0.0025	0.0152
Lancaster K.J.	2	0.0000	0.0024	0.0157

**Table 8:** Composition of co-citation network 3 in terms of authors, articles, year, country of authors and citation.

Year	#	Authors	Articles	Year	Country of Authors	Citation
2011–2016	1	Roberto, C. A., Bragg, M. A., Schwartz, M. B., Seamans, M. J., Musicus, A., Novak, N., & Brownell, K. D.	Facts Up Front Versus Traffic Light Food Labels	2012	USA	156
	2	Signal, L., Lanumata, T., Mhurchu, C. N., & Gorton, D.	Front-of-pack nutrition labelling in New Zealand: an exploration of stakeholder views about research and implementation	2012	USA	5
	3	Storcksdieck genannt Bonsmann, S., & Wills, J. M.	Nutrition Labeling to Prevent Obesity: Reviewing the Evidence from Europe	2012	Belgium	71
	4	Eric van Herpen, Ellen Seissb, Hans C.M. Van Trijpc	The role of familiarity in front-of-pack label evaluation and use: A comparison between the United Kingdom and The Netherlands	2012	The Netherlands	61
	5	Hodgkins, C., Barnett, J., Wasowicz-Kirylo, G., Stysko-Kunkowska, M., Gulcan, Y., Kustepeli, Y., ... & Raats, M.	Understanding how consumers categorise nutritional labels: A consumer derived typology for front-of-pack nutrition labelling	2012	UK, Poland, Turkey, Belgium, Greece	169
	6	Aschemann-Witzel, J., Grunert, K. G., van Trijp, H. C., Bialkova, S., Raats, M. M., Hodgkins, C., ... & Koenigstorfer, J.	Effects of nutrition label format and product assortment on the healthfulness of food choice	2013	The Netherlands, UK, Poland, Germany	183
	7	M. Rayner, A. Wood, M. Lawrence, C. N. Mhurchu, J. Albert, S. Barquera, S. Friel, C. Hawkes, B. Kelly, S. Kumanyika, M. L’Abbé, A. Lee, T. Lobstein, J. Ma, J. Macmullan, S. Mohan, C. Monteiro, B. Neal, G. Sacks, D. Sanders, W. Snowdon, B. Swinburn, S. Vandevijvere and C. Walker	Monitoring the health-related labelling of foods and non-alcoholic beverages in retail settings	2013	UK, New Zealand, Italy, Australia, Canada, Switzerland	121
	8	Hawley, K. L., Roberto, C. A., Bragg, M. A., Liu, P. J., Schwartz, M. B., & Brownell, K. D.	The science on front-of-package food labels	2013	USA	497
	9	Mejean, C., Macouillard, P., Péneau, S., Hercberg, S., & Castetbon, K.	Consumer acceptability and understanding of front-of-pack nutrition labels	2013	France	85
	10	Hersey, J. C., Wohlgenant, K. C., Arsenault, J. E., Kosa, K. M., & Muth, M. K.	Effects of front-of-package and shelf nutrition labeling systems on consumers	2013	USA	369
	11	Mejean, C., Macouillard, P., Péneau, S., Hercberg, S., & Castetbon, K.	Perception of front-of-pack labels according to social characteristics, nutritional knowledge and food purchasing habits	2013	France	84
	12	Andrews, J. C., Lin, C. T. J., Levy, A. S., & Lo, S.	Consumer Research Needs from the Food and Drug Administration on Front-of-Package Nutritional Labeling	2014	USA	81
	13	Van Herpen, E., Hieke, S., & Van Trijp, H. C.	Inferring product healthfulness from nutrition labelling. The influence of reference points	2014	The Netherlands, Germany	58
	14	Devi, A., Eyles, H., Rayner, M., Mhurchu, C. N., Swinburn, B., Lonsdale-Cooper, E., & Vandevijvere, S.	Nutritional quality, labelling and promotion of breakfast cereals on the New Zealand market	2014	New Zealand, UK, Australia	74
	15	Méjean, C., Macouillard, P., Péneau, S., Lassale, C., Hercberg, S., & Castetbon, K.	Association of Perception of Front-of-Pack Labels with Dietary, Lifestyle and Health Characteristics	2014	France	35
	16	Volkova, E., Neal, B., Rayner, M., Swinburn, B., Eyles, H., Jiang, Y., ... & Ni Mhurchu, C	Effects of interpretive front-of-pack nutrition labels on food purchases: protocol for the Starlight randomised controlled trial	2014	New Zeland	23
	17	Julia, C., Ducrot, P., Péneau, S., Deschamps, V., Méjean, C., Fézeu, L., ... & Kesse-Guyot, E.	Discriminating nutritional quality of foods using the 5-Color nutrition label in the French food market: consistency with nutritional recommendations	2015	France	64
	18	Ducrot, P., Julia, C., Méjean, C., Kesse-Guyot, E., Touvier, M., Fezeu, L. K., ... & Péneau, S.	Objective Understanding of Front-of-Package Nutrition Labels among Nutritionally At-Risk Individuals	2015	France	98
	19	Julia, C., Touvier, M., Méjean, C., Ducrot, P., Péneau, S., Hercberg, S., & Kesse-Guyot, E.	Performance of a five category front-of-pack labelling system—the 5-colour nutrition label—to differentiate nutritional quality of breakfast cereals in France	2015	France	71
	20	Ducrot, P., Julia, C., Méjean, C., Kesse-Guyot, E., Touvier, M., Fezeu, L. K., ... & Péneau, S.	Impact of Different Front-of-Pack Nutrition Labels on Consumer Purchasing Intentions A Randomized Controlled Trial	2016	France	182
	21	Julia, C., Blanchet, O., Méjean, C., Péneau, S., Ducrot, P., Allès, B., ... & Hercberg, S.	Impact of the front-of-pack 5-colour nutrition label (5-CNL) on the nutritional quality of purchases: an experimental study	2016	France	82
	22	Fenko, A., Kersten, L., & Bialkova, S.	Overcoming consumer skepticism toward food labels: The role of multisensory experience	2016	The Netherlands	96
	23	Hieke, S., Kuljanic, N., Pravst, I., Miklavec, K., Kaur, A., Brown, K. A., ... & Rayner, M.	Prevalence of Nutrition and Health-Related Claims on Pre-Packaged Foods: A Five-Country Study in Europe	2016	Belgium, Spain, Germany, Slovenia	90
	24	Julia, C., Méjean, C., Péneau, S., Buscail, C., Alles, B., Fezeu, L., ... & Kesse-Guyot, E.	The 5-CNL Front-of-Pack Nutrition Label Appears an Effective Tool to Achieve Food Substitutions towards Healthier Diets across Dietary Profiles	2016	France	23

**Table 9:** The relative strength of the nodes within co-citation network 3.

Node	Co-Citation Network	Betweenness	Closeness	PageRank
Julia C.	3	0.4858	0.0071	0.0191
Hercberg S.	3	1.4783	0.0074	0.0201
Rayner M.	3	1.4783	0.0074	0.0201
Feunekes G.I.	3	280.0777	0.0094	0.0213
Vyth E.L.	3	1.4185	0.0074	0.0185
Cowburn G.	3	184.7231	0.0093	0.0203
Food Standards Agency	3	9.4425	0.0078	0.0196
Hawley K.L.	3	78.2377	0.0085	0.0208
Mejean C.	3	0.9160	0.0074	0.0185
Bialkova S.	3	10.7832	0.0078	0.0184
Bonsmann S.S.G.	3	16.5462	0.0078	0.0181
Campos S.	3	73.9718	0.0083	0.0199
Hersey J.C.	3	24.1884	0.0082	0.0197
Sacks G.	3	1.1089	0.0072	0.0184
Who	3	17.9771	0.0077	0.0191
Borgmeier I.	3	8.7401	0.0075	0.0184
Van Herpen E.	3	15.0560	0.0082	0.0193
Ducrot P.	3	0.0216	0.0068	0.0161
Gorton D.	3	45.7196	0.0079	0.0178
Schwartz M.B.	3	15.8411	0.0071	0.0165

**Table 10:** Composition of co-citation network 4 in terms of authors, articles, year, country of authors and citation.

	Id	Authors	Articles	Year	Country of Authors	Citation
2012–2016	1	Liu, P. J., Roberto, C. A., Liu, L. J., & Brownell, K. D.	A test of different menu labeling presentations	2012	USA	139
	2	Roberto, C. A., Bragg, M. A., Livingston, K. A., Harris, J. L., Thompson, J. M., Seamans, M. J., & Brownell, K. D.	Choosing front-of-package food labelling nutritional criteria: how smart were ‘Smart Choices’?	2012	USA	39
	3	Roberto, C. A., Bragg, M. A., Schwartz, M. B., Seamans, M. J., Musicus, A., Novak, N., & Brownell, K. D.	Facts Up Front Versus Traffic Light Food Labels A Randomized Controlled Trial	2012	USA	158
	4	Roberto, C. A., Shivaram, M., Martinez, O., Boles, C., Harris, J. L., & Brownell, K. D.	The Smart Choices front-of-package nutrition label. Influence on perceptions and intake of cereal	2012	USA	104
	5	Moorman, C., Ferraro, R., & Huber, J.	Unintended Nutrition Consequences: Firm Responses to the Nutrition Labeling and Education Act	2012	USA	91
	6	Roberto, C. A., Bragg, M. A., Seamans, M. J., Mechulan, R. L., Novak, N., & Brownell, K. D.	Evaluation of Consumer Understanding of Different Front-of-Package Nutrition Labels, 2010–2011	2012	USA	68
	7	Sacks, G., Swinburn, B., Kraak, V., Downs, S., Walker, C., Barquera, S., ... & INFORMAS.	A proposed approach to monitor private-sector policies and practices related to food environments, obesity and non-communicable disease prevention	2013	Australia, New Zealand, USA, UK	84
	8	Martinez, O. D., Roberto, C. A., Kim, J. H., Schwartz, M. B., & Brownell, K. D.	A Survey of undergraduate student perceptions and use of nutrition information labels in a university dining hall	2013	USA	53
	9	Aschemann-Witzel, J., Grunert, K. G., van Trijp, H. C., Bialkova, S., Raats, M. M., Hodgkins, C., ... & Koenigstorfer, J.	Effects of nutrition label format and product assortment on the healthfulness of food choice	2013	Denmark, The Netherlands, UK, Poland, Germany	183
	10	Wendy L Watson, Kathy Chapman, Lesley King, Bridget Kelly, Clare Hughes, Jimmy Chun Yu Louie, Jennifer Crawford and Timothy P Gill	How well do Australian shoppers understand energy terms on food labels?	2013	Australia	49
	11	Joseph, S., Lavoie, N., & Caswell, J. A.	Implementing COOL: Comparative welfare effects of different labeling schemes	2013	USA	13
	12	Rayner, M., Wood, A., Lawrence, M., Mhurchu, C. N., Albert, J., Barquera, S., ... & INFORMAS.	Monitoring the health-related labelling of foods and non-alcoholic beverages in retail settings	2013	UK, Italy, USA	122
	13	Bialkova, S., Grunert, K. G., & van Trijp, H.	Standing out in the crowd: The effect of information clutter on consumer attention for front-of-pack nutrition labels	2013	Denmark, The Netherlands	111
	14	Hawley, K. L., Roberto, C. A., Bragg, M. A., Liu, P. J., Schwartz, M. B., & Brownell, K. D.	The science on front-of-package food labels	2013	USA	497
	15	Lee, W. C. J., Shimizu, M., Kniffin, K. M., & Wansink, B	You taste what you see: Do organic labels bias taste perceptions?	2013	USA	387
	16	Bialkova, S., Grunert, K. G., Juhl, H. J., Wasowicz-Kirylo, G., Stysko-Kunkowska, M., & van Trijp, H. C.	Attention mediates the effect of nutrition label information on consumers’ choice. Evidence from a choice experiment involving eye-tracking	2014	Denmark, The Netherlands, Poland	155
	17	Watson, W. L., Kelly, B., Hector, D., Hughes, C., King, L., Crawford, J., ... & Chapman, K.	Can front-of-pack labelling schemes guide healthier food choices? Australian shoppers’ responses to seven labelling formats	2014	Australia	127
	18	Dixon, H., Scully, M., Kelly, B., Donovan, R., Chapman, K., & Wakefield, M.	Counter-Advertising May Reduce Parent’s Susceptibility to Front-of-Package Promotions on Unhealthy Foods	2014	Australia	23
	19	Roberto, C. A., & Khandpur, N.	Improving the design of nutrition labels to promote healthier food choices and reasonable portion sizes	2014	USA	152
	20	Newman, C. L., Howlett, E., & Burton, S.	Shopper Response to Front-of-Package Nutrition Labeling Programs: Potential Consumer and Retail Store Benefits	2014	USA	107
	21	Grunert, K. G., Hieke, S., & Wills, J.	Sustainability labels on food products: Consumer motivation, understanding and use	2014	Denmark, Belgium	1050
	22	Andrews, J. C., Lin, C. T. J., Levy, A. S., & Lo, S.	Consumer Research Needs from the Food and Drug Administration on Front-of-Package Nutritional Labeling	2014	USA	81
	23	Kauer, J., Pelchat, M. L., Rozin, P., & Zickgraf, H. F.	Adult picky eating. Phenomenology, taste sensitivity, and psychological correlates	2015	USA	122
	24	Hodgkins, C. E., Raats, M. M., Fife-Schaw, C., Peacock, M., Gröppel-Klein, A., Koenigstorfer, J., ... & Grunert, K. G.	Guiding healthier food choice: systematic comparison of four front-of-pack labelling systems and their effect on judgements of product healthiness	2015	UK, Poland, Turkey, Denmark	62
	25	Kleef, E. V., & Dagevos, H.	The Growing Role of Front-of-Pack Nutrition Profile Labeling: A Consumer Perspective on Key Issues and Controversies	2015	The Neterlands	214
	26	Wąsowicz, G., Styśko-Kunkowska, M., & Grunert, K. G.	The meaning of colours in nutrition labelling in the context of expert and consumer criteria of evaluating food product healthfulness	2015	Poland, Denmark	27
	27	Grunert, K. G., & Aachmann, K.	Consumer reactions to the use of EU quality labels on food products: A review of the literature	2016	Denmark	195
	28	Talati, Z., Pettigrew, S., Kelly, B., Ball, K., Dixon, H., & Shilton, T.	Consumers’ responses to front-of-pack labels that vary by interpretive content	2016	Australia	73
	29	Newman, C. L., Howlett, E., & Burton, S.	Effects of Objective and Evaluative Front-of-Package Cues on Food Evaluation and Choice: The Moderating Influence of Comparative and Noncomparative Processing Contexts	2016	UK	61
	30	VanEpps, E. M., Roberto, C. A., Park, S., Economos, C. D., & Bleich, S. N.	Restaurant Menu Labeling Policy: Review of Evidence and Controversies	2016	USA	102
	31	Talati, Z., Pettigrew, S., Hughes, C., Dixon, H., Kelly, B., Ball, K., & Miller, C.	The combined effect of front-of-pack nutrition labels and health claims on consumers’ evaluation of food products	2016	Australia	51
	32	Qi, D., & Roe, B. E.	Household Food Waste: Multivariate Regression and Principal Components Analyses of Awareness and Attitudes among U.S. Consumers	2016	USA	205

**Table 11:** The relative strength of the nodes within co-citation network 4.

Node	Co-Citation Network	Betweenness	Closeness	PageRank
Wansink B.	4	75.6265	0.0088	0.0276
Grunert K.G.	4	248.3855	0.0100	0.0261
Roberto C.A.	4	62.7962	0.0089	0.0202
Moorman C.	4	4.4328	0.0072	0.0212
Andrews J.C.	4	2.1948	0.0070	0.0209
Kozup J.C.	4	15.3196	0.0081	0.0253
Burton S.	4	0.6517	0.0064	0.0229
Chandon P.	4	0.0000	0.0064	0.0195
Keller S.B.	4	6.2964	0.0076	0.0240
Rozin P.	4	0.0000	0.0031	0.0171
Viswanathan M.	4	2.1404	0.0068	0.0197
Kelly B.	4	99.2021	0.0094	0.0226
Van Kleef E.	4	100.5196	0.0094	0.0223
Caswell J.A.	4	49.2315	0.0070	0.0277
Petty R.E.	4	0.0000	0.0058	0.0163
Drewnowski A.	4	0.0000	0.0032	0.0202
Drichoutis A.C.	4	3.7479	0.0072	0.0190
Levy A.S.	4	6.7481	0.0078	0.0191
Roe B.	4	5.4736	0.0078	0.0204
Nestle M.	4	2.2088	0.0068	0.0194
Finkelstein E.A.	4	4.4040	0.0063	0.0202
Food Marketing Institute	4	0.0000	0.0026	0.0151
Harris J.L.	4	0.9498	0.0060	0.0189
Just D.R.	4	0.0000	0.0029	0.0163
Nayga R.M.	4	0.0000	0.0030	0.0169
Verbeke W.	4	2.6675	0.0069	0.0197
Williams P.	4	0.2657	0.0065	0.0180
Flegal K.M.	4	4.5540	0.0066	0.0208

**Table 12:** Composition of co-citation network 4 in terms of authors, articles, year, country of authors and citation.

	Id	Authors	Articles	Year	Country of Authors	Citation
2017–2022	1	Zhang, Y., Chen, J. T., Wang, S., Andrews, J. C., & Levy, A.	How Do Consumers Use Nutrition Labels on Food Products in the United States?	2017	USA	8
	2	Findling, M. T. G., Werth, P. M., Musicus, A. A., Bragg, M. A., Graham, D. J., Elbel, B., & Roberto, C. A	Comparing five front-of-pack nutrition labels’ influence on consumers’ perceptions and purchase intentions	2018	USA	75
	3	Menger-Ogle, A. D., & Graham, D. J.	The influence of front-of-package nutrition claims on food perceptions and purchase intentions among Nepali consumers	2018	USA	13
	4	Rybak, G., Burton, S., Johnson, A. M., & Berry, C.	Promoted claims on food product packaging: Comparing direct and indirect effects of processing and nutrient content claims	2021	USA	2
	5	Meijer, G. W., Detzel, P., Grunert, K. G., Robert, M. C., & Stancu, V.	Towards effective labelling of foods. An international perspective on safety and nutrition	2021	USA	3
	6	Andrews, J. C., Netemeyer, R., Burton, S., & Kees, J.	What consumers actually know: The role of objective nutrition knowledge in processing stop sign and traffic light front-of-pack nutrition labels	2021	USA	5

**Table 13:** The relative strength of the nodes within the co-citation network 4.

Node	Co-Citation Network	Betweenness	Closeness	PageRank
Grunert KG	4	184.6549	0.0164	0.0220
Bialkova S	4	19.1945	0.0152	0.0217
Cowburn G	4	7.4175	0.0147	0.0211
Graham DJ	4	6.2105	0.0147	0.0204
Newman CL	4	3.7383	0.0141	0.0204
Sutherland LA	4	3.0255	0.0133	0.0208
Drichoutis AC	4	2.8982	0.0120	0.0196
Andrews JC	4	1.9661	0.0133	0.0213
Roberto CA	4	1.4009	0.0128	0.0199
Moorman C	4	0.4905	0.0110	0.0192
Wansink B	4	0.2577	0.0111	0.0185
Burton S	4	0.0292	0.0109	0.0194
Chandon P	4	0.0078	0.0100	0.0196

**Table 14:** Composition of co-citation network 5 in terms of authors, articles, year, country of authors and citation.

	Id	Authors	Articles	Year	Country of Authors	Citation
2017–2022	1	Talati, Z., Norman, R., Kelly, B., Dixon, H., Neal, B., Miller, C., & Pettigrew, S.	A randomized trial assessing the effects of health claims on choice of foods in the presence of front-of-pack labels	2018	Australia	16
	2	Talati, Z., Pettigrew, S., Kelly, B., Ball, K., Neal, B., Dixon, H., ... & Miller, C.	Can front-of-pack labels influence portion size judgements for unhealthy foods?	2018	Australia	9
	3	Acton, R. B., Vanderlee, L., & Hammond, D.	Influence of front-of-package nutrition labels on beverage healthiness T perceptions: Results from a randomized experiment	2018	USA	18
	4	Egnell, M., Talati, Z., Hercberg, S., Pettigrew, S., & Julia, C.	Objective Understanding of Front-of-Package Nutrition Labels: An International Comparative Experimental Study across 12 Countries	2018	France	162
	5	Dana, L. M., Chapman, K., Talati, Z., Kelly, B., Dixon, H., Miller, C., & Pettigrew, S.	Consumers’ Views on the Importance of Specific Front-of-Pack Nutrition Information: A Latent Profile Analysis	2019	Australia	11
	6	Talati, Z., Egnell, M., Hercberg, S., Julia, C., & Pettigrew, S.	Food Choice Under Five Front-of-Package Nutrition Label Conditions: An Experimental Study Across 12 Countries	2019	France	29
	7	Kelly, B., & Jewell, J.	Front-of-pack nutrition labelling in the European region: identifying what works for governments and consumers	2019	Denemark	9

**Table 15:** Correlation analysis with network measures.

Variable	Betweenness Centrality	Closeness Centrality	PageRank
Betweenness centrality			
Closeness centrality	0.47 ** [0.34, 0.58]		
PageRank	0.47 ** [0.35, 0.58]	0.55 ** [0.44, 0.65]	
H-index	0.14 ** [0.9, 0.19]	0.18 ** [0.11, 0.23]	0.24 ** [0.09, 0.37]

** = *p* < 0.01.

**Table 16:** Position of authors in the network and their measures. Authors in bold are those with a value above the mean.

Author	Betweenness	Closeness	PageRank	H_Index
Julia C.	5046.0840	0.00005709	0.0100	15
Hercberg S.	2630.1252	0.00005682	0.0068	14
Talati Z.	2337.1708	0.00005735	0.0058	10
Pettigrew S.	1917.2877	0.00005733	0.0024	10
Touvier M.	152.3228	0.00005653	0.0013	10
Burton S.	4048.7644	0.00005739	0.0089	8
Egnell M.	2123.5205	0.00005680	0.0061	8
Mejean C.	942.5946	0.00005687	0.0053	7
Dixon H.	18.9825	0.00005692	0.0017	7
Kelly B.	8436.2534	0.00005788	0.0053	6
Miller J.C.	0.0000	0.00004584	0.0009	6
Galan P.	0.0000	0.00005628	0.0004	6
Ducrot P.	3603.5131	0.00005722	0.0080	5
Howlett E.	767.1588	0.00005673	0.0031	5
Newman C.L.	5421.8157	0.00005766	0.0035	4
Andrews J.C.	5074.1672	0.00005750	0.0113	3
Deschasaux M.	90.0606	0.00005634	0.0014	3
Kees J.	115.9491	0.00005656	0.0007	3
Hughes C.	0.0000	0.00005524	0.0003	3

**Table 17:** Average betweenness, closeness centrality and PageRank per co-citation network compared to the average mean of the network.

	Betweenness Centrality (Avg)	Closeness Centrality (Avg)	PageRank (Avg)
Mean of co-citation network 1	37.8750	0.0039	0.0234
Mean of co-citation network 2	22.0833	0.0040	0.0223
Mean of co-citation network 3	39.4106	0.0078	0.0190
Mean of co-citation network 4	28.8621	0.0067	0.0208
Mean of co-citation network 6	17.7916	0.0130	0.0203
Mean of co-citation network 5	19.8293	0.0132	0.0194
Mean of co-citation network 7	9.6941	0.0126	0.0203
Overall Mean	25.0780	0.0088	0.0208

**Table 18:** Evolution of Composition of co-citation network 3 in terms of authors, articles, year, country of authors and citation.

	Id	Authors	Articles	Year	Country of Authors	Citation
2017–2022	1	Egnell, M., Ducrot, P., Touvier, M., Allès, B., Hercberg, S., Kesse-Guyot, E., & Julia, C.	Objective understanding of Nutri-Score Front-Of-Package nutrition label according to individual characteristics of subjects: Comparisons with other format labels	2018	France	91
	2	Egnell, M., Boutron, I., Péneau, S., Ducrot, P., Touvier, M., Galan, P., ... & Julia, C.	Front-of-Pack Labeling and the Nutritional Quality of Students’ Food Purchases: A 3-Arm Randomized Controlled Trial	2019	France	26
	3	Egnell, M., Crosetto, P., D’almeida, T., Kesse-Guyot, E., Touvier, M., Ruffieux, B., ... & Julia, C.	Modelling the impact of different front-of- package nutrition labels on mortality from non-communicable chronic disease	2019	France	50
	4	Crosetto, P., Lacroix, A., Muller, L., & Ruffieux, B.	Nutritional and economic impact of five alternative front-of-pack nutritional labels: experimental evidence	2020	France	43
	5	Talati, Z., Egnell, M., Hercberg, S., Julia, C., & Pettigrew, S.	Consumers’ Perceptions of Five Front-of-Package Nutrition Labels: An Experimental Study Across 12 Countries	2020	France	49
	6	Fialon, M., Egnell, M., Talati, Z., Galan, P., Dréano-Trécant, L., Touvier, M., ... & Julia, C.	Effectiveness of Different Front-of-Pack Nutrition Labels among Italian Consumers: Results from an Online Randomized Controlled Trial	2020	France	17
	7	Dréano-Trécant, L., Egnell, M., Hercberg, S., Galan, P., Soudon, J., Fialon, M., ... & Julia, C.	Performance of the Front-of-Pack Nutrition Label Nutri-Score to Discriminate the Nutritional Quality of Foods Products: A Comparative Study across 8 European Countries	2020	France	39
	8	Sarda, B., Julia, C., Serry, A. J., & Ducrot, P.	Appropriation of the Front-of-Pack Nutrition Label Nutri-Score across the French Population: Evolution of Awareness, Support, and Purchasing Behaviors between 2018 and 2019	2020	France	11

**Table 19:** Evolution of the relative strength of the nodes within the co-citation network 3.

Node	Co-Citation Network	Betweenness	Closeness	PageRank
Julia C.	3	23.0186	0.0149	0.0199
Egnell M.	3	21.1437	0.0147	0.0198
Ducrot P.	3	33.2542	0.0149	0.0199
Hersey J.C.	3	126.8358	0.0159	0.0203
Crosetto P.	3	12.9291	0.0143	0.0196
Hawley K.L.	3	33.9948	0.0152	0.0200
Vyth E.L.	3	23.4873	0.0149	0.0196
Who	3	9.1579	0.0143	0.0196
Hercberg S.	3	0.5694	0.0116	0.0189
Chantal J.	3	10.0508	0.0143	0.0196
Adriouch S.	3	0.0358	0.0106	0.0186
Rayner M.	3	0.2922	0.0108	0.0186
Deschasaux M.	3	0.0000	0.0101	0.0183
Donnenfeld M.	3	0.0358	0.0106	0.0186
Dubois P.	3	2.6335	0.0115	0.0189

## Data Availability

Not applicable.

## Supplementary Materials

The following are available online at https://www.mdpi.com/article/10.3390/nu14163423/s1, Figure S1: 1989–2011: Discovering the relevance of nutrition-related information; Figure S2: 2012–2016—The drivers of acceptance of Front-of-Pack labels; Figure S3: Period: 2017–2022—Opening up to alternatives and challenging the mainstream.
