# Supplementary material for: Uncovering the Effect of European Policy-Making Initiatives in Addressing Nutrition-Related Issues: A Systematic Literature Review and Bibliometric Analysis on Front-of-Pack Labels
Source: Nutrients. 2022 Aug 19;14(16):3423. doi: 10.3390/nu14163423 (PMC9414449; doi:10.3390/nu14163423)
Supplement: Supplementary file 1 [file nutrients-14-03423-s001.zip › nutrients-1819190-supplementary.pdf]

SUPPLEMENTARY MATERIALS: Uncovering the Effect of European Policy-Making Initiatives in Addressing Nutrition-Related Issues: A Systematic Literature Review and Bibliometric Analysis on Front-of-Pack Labels

Figure S1. 1989-2011: Discovering the relevance of nutrition-related information.

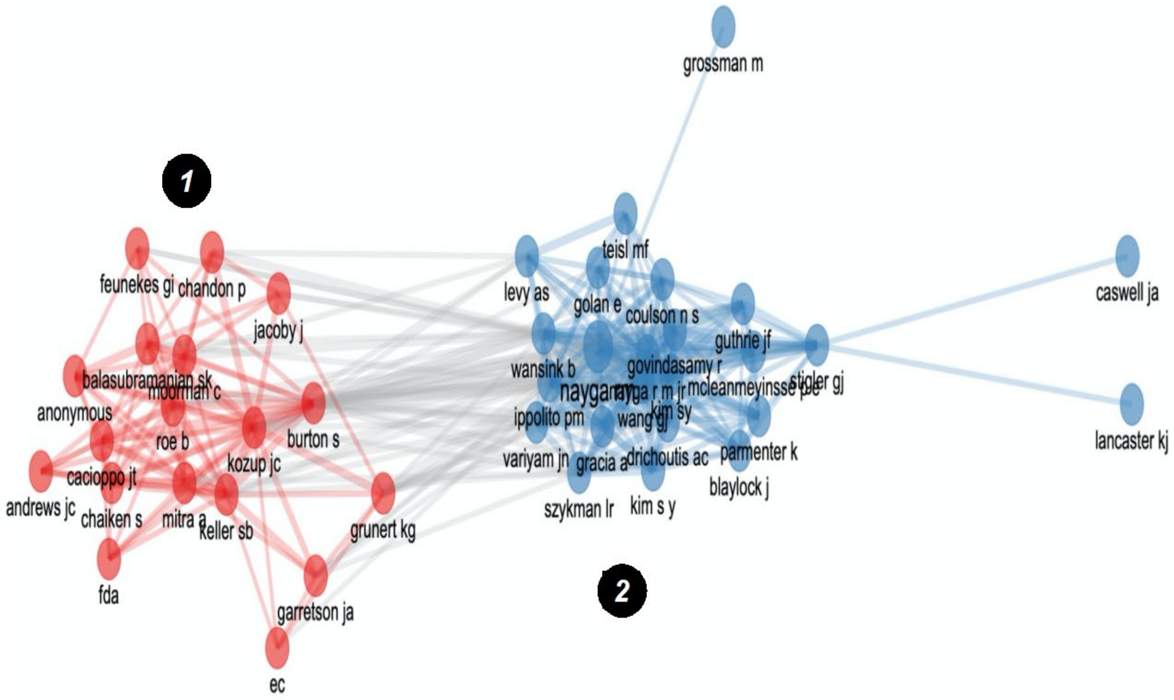

1989-2011: Discovering the relevance of nutrition-related information.

Figure S2. 2012 – 2016 - The drivers of acceptance of Front-of-Pack labels

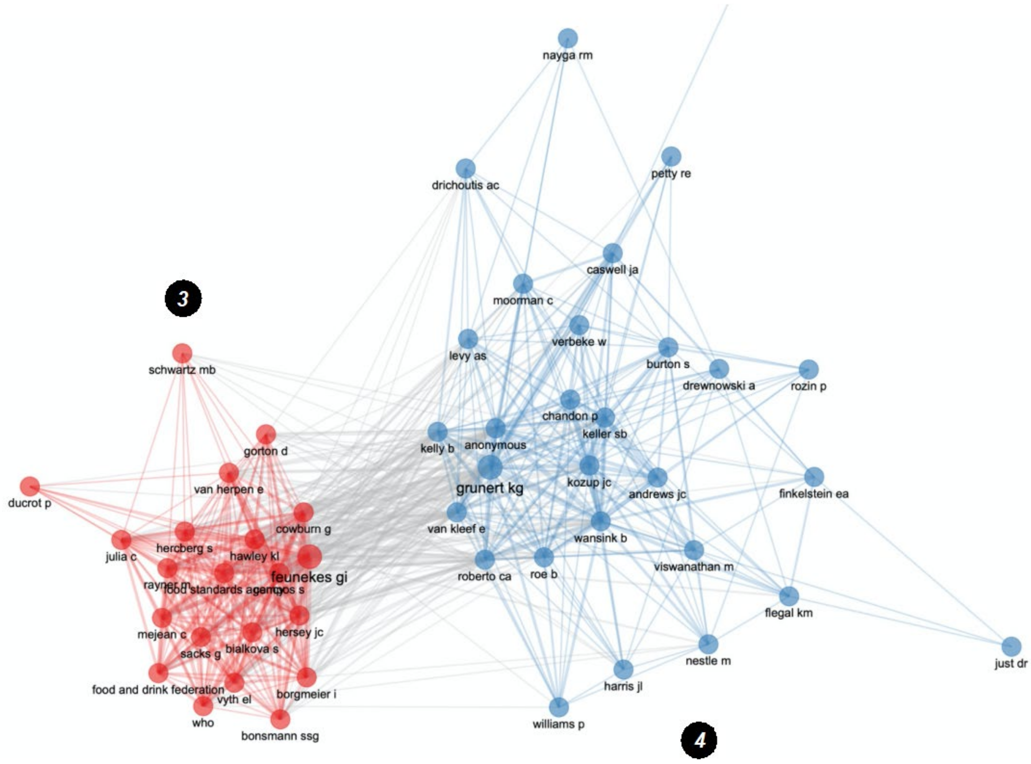

2012 – 2016 - The drivers of acceptance of Front-of-Pack labels.

Figure S3. Period: 2017 – 2022 – Opening up to alternatives and challenging the mainstream

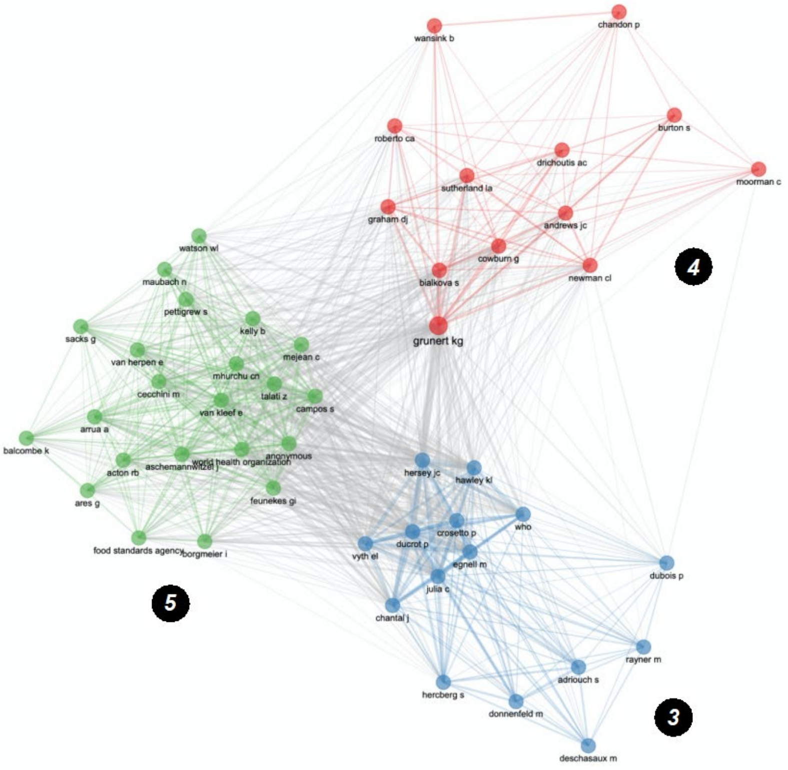

Period: 2017 – 2022 – Opening up to alternatives and challenging the mainstream.
